# SMOTE for high-dimensional class-imbalanced data

**DOI:** 10.1186/1471-2105-14-106

**Published:** 2013-03-22

**Authors:** Rok Blagus, Lara Lusa

**Affiliations:** 1 Institute for Biostatistics and Medical Informatics, University of Ljubljana, Vrazov trg 2, Ljubljana, Slovenia

## Abstract

**Background:**

Classification using class-imbalanced data is biased in favor of the majority class. The bias is even larger for high-dimensional data, where the number of variables greatly exceeds the number of samples. The problem can be attenuated by undersampling or oversampling, which produce class-balanced data. Generally undersampling is helpful, while random oversampling is not. Synthetic Minority Oversampling TEchnique (SMOTE) is a very popular oversampling method that was proposed to improve random oversampling but its behavior on high-dimensional data has not been thoroughly investigated. In this paper we investigate the properties of SMOTE from a theoretical and empirical point of view, using simulated and real high-dimensional data.

**Results:**

While in most cases SMOTE seems beneficial with low-dimensional data, it does not attenuate the bias towards the classification in the majority class for most classifiers when data are high-dimensional, and it is less effective than random undersampling. SMOTE is beneficial for k-NN classifiers for high-dimensional data if the number of variables is reduced performing some type of variable selection; we explain why, otherwise, the k-NN classification is biased towards the minority class. Furthermore, we show that on high-dimensional data SMOTE does not change the class-specific mean values while it decreases the data variability and it introduces correlation between samples. We explain how our findings impact the class-prediction for high-dimensional data.

**Conclusions:**

In practice, in the high-dimensional setting only k-NN classifiers based on the Euclidean distance seem to benefit substantially from the use of SMOTE, provided that variable selection is performed before using SMOTE; the benefit is larger if more neighbors are used. SMOTE for k-NN without variable selection should not be used, because it strongly biases the classification towards the minority class.

## Background

The objective of class prediction (classification) is to develop a rule based on a group of samples with known class membership (training set), which can be used to assign the class membership to new samples. Many different classification algorithms (classifiers) exist, and they are based on the values of the variables (features) measured for each sample [1].

Very often the training and/or test data are class-imbalanced: the number of observations belonging to each class is not the same. The problem of learning from class-imbalanced data has been receiving a growing attention in many different fields [2]. The presence of class-imbalance has important consequences on the learning process, usually producing classifiers that have poor predictive accuracy for the minority class and that tend to classify most new samples in the majority class; in this setting the assessment of the performance of the classifiers is also critical [3].

Data are nowadays increasingly often high-dimensional: the number of variables is very large and greatly exceeds the number of samples. For example, high-throughput technologies are popular in the biomedical field, where it is possible to measure simultaneously the expression of all the known genes (>20,000) but the number of subjects included in the study is rarely larger than few hundreds. Many papers attempted to develop classification rules using high-dimensional gene expression data that were class-imbalanced (see for example [4-6]).

Despite the growing number of applications using high-dimensional class-imbalanced data, this problem has been seldom addressed from the methodological point of view [2]. It was previously shown for many classifiers that the class-imbalance problem is exacerbated when data are high-dimensional [7]: the high-dimensionality further increases the bias towards the classification into the majority class, even when there is no real difference between the classes. The high-dimensionality affects each type of classifier in a different way. A general remark is that large discrepancies between training data and true population values are more likely to occur in the minority class, which has a larger sampling variability: therefore, the classifiers are often trained on data that do not represent well the minority class. The high-dimensionality contributes to this problem as extreme values are not exceptional when thousands of variables are considered.

Some of the solutions proposed in the literature to attenuate the class-imbalance problem are effective with high-dimensional data, while others are not. Generally undersampling techniques, aimed at producing a class-balanced training set of smaller size, are helpful, while simple oversampling is not [7]. The reason is that in most cases simple oversampling does not change the classification rule. Similar results were obtained also for low-dimensional data [8].

The Synthetic Minority Over-sampling TEchnique (SMOTE [9]) is an oversampling approach that creates synthetic minority class samples. It potentially performs better than simple oversampling and it is widely used. For example, SMOTE was used for detecting network intrusions [10] or sentence boundary in speech [11], for predicting the distribution of species [12] or for detecting breast cancer [13]. SMOTE is used also in bioinformatics for *miRNA* gene prediction [14,15], for the identification of the binding specificity of the regulatory proteins [16] and of photoreceptor-enriched genes based on expression data [17], and for histopathology annotation [18].

However, it was recently experimentally observed using low-dimensional data that simple undersampling tends to outperform SMOTE in most situations [8]. This result was further confirmed using SMOTE with SVM as a base classifier [19], extending the observation also to high-dimensional data: SMOTE with SVM seems beneficial but less effective than simple undersampling for low-dimensional data, while it performs very similarly to uncorrected SVM and generally much worse than undersampling for high-dimensional data. To our knowledge this was the first attempt to investigate explicitly the effect of the high-dimensionality on SMOTE, while the performance of SMOTE on high-dimensional data was not thoroughly investigated for classifiers other than SVM. Others evaluated the performance of SMOTE on large data sets, focusing on problems where the number of samples, rather than the number of variables was very large [20,21]. A number of works focused on improving the original SMOTE algorithm [17,22-24] but these modifications were mainly not considered in the high-dimensional context.

In this article we investigate the theoretical properties of SMOTE and its performance on high-dimensional data. For the sake of simplicity we consider only two-class classification problems, and limit our attention to Classification and Regression Trees (CART [25]), k-NN [26] with *k* = 1, 3 and 5, linear discriminant analysis methods (diagonal - DLDA, and quadratic - DQDA) [27,28], random forests (RF [29]), support vector machine (SVM [30]), prediction analysis for microarrays (PAM [31] also known as nearest shrunken centroids classification) and penalized logistic regression (PLR [32]) with the linear (PLR-L1) and quadratic penalty (PLR-L2). We supplement the theoretical results with empirical results, based on simulation studies and analysis of gene expression microarray data sets.

The rest of the article is organized as follows. In the Results Section we present some theoretical results, a selected series of simulation results and the experimental results. In the Discussion Section we summarize and discuss the most important results of our study. In the Methods Section we briefly describe SMOTE and simple undersampling, the classification algorithms, the variable selection method and the performance measures that we used; we also describe the procedure of data simulation, the breast cancer gene expression data sets and the classification problems addressed.

## Results

In this section we present some theoretical properties of SMOTE [9], the simulation results and the experimental data results.

SMOTE is an oversampling technique that generates synthetic samples from the minority class. It is used to obtain a synthetically class-balanced or nearly class-balanced training set, which is then used to train the classifier. The SMOTE samples are linear combinations of two similar samples from the minority class (**x** and **x**^*R*^) and are defined as

(1) $$\text{s}=\text{x}+u\cdot ({\text{x}}^{R}-\text{x}),$$

with 0 ≤ *u* ≤ 1; **x**^*R*^ is randomly chosen among the 5 minority class nearest neighbors of **x**. We refer the reader to the Methods section for a more detailed description of the method and of the notation used in the paper.

### Theoretical properties of SMOTE for high-dimensional data

In this section we present some theoretical properties of SMOTE for high-dimensional data, which are summarized in Table 1.

**Table 1:** Summary of the theoretical properties of SMOTE for high-dimensional data

**Property**	**Consequence of using SMOTE on high-dimensional data**
*E*(SMOTE) = *E*(*X*)	Little impact on classifiers that depend on mean values (DLDA);
$\text{var}(\text{SMOTE})=\frac{2}{3}\text{var}(X)$	Minority class variability is underestimated; negative impact on classifiers that use class-specific variances (DQDA); inflated statistical significance of statistical tests for comparing classes (t-test);
*d*(SMOTE, TEST) < *d*(*X*, TEST)*d*: Euclidean distance	Test samples are classified mostly in the minority class for classifiers based on Euclidean distance (k-NN); variable selection is helpful in reducing this problem;
*cor*(SMOTE, *X*) ≥ 0; *cor*(SMOTE^*s*^, SMOTE^*t*^) ≥ 0	Training set samples are no longer independent; independence of samples is assumed by most classifiers (DLDA, PLR,...) and variable selection methods (t-test, Mann-Whitney,...)

Most of the proofs require the assumptions that **x**^*R*^ and **x** are independent and have the same expected value (*E*(·)) and variance (*var*(·)). We conducted a limited set of simulations in which we showed that in practice these assumptions are valid for high-dimensional data, while they do not hold for low-dimensional data (Additional file 1), where the samples are positively correlated. Similar results were described by others [33,34].

The proofs and details of the results presented in this section are given in Additional file 1, where most of the results are derived also without assuming the independence and equal distribution of the original and nearest neighbor samples.

#### SMOTE does not change the expected value of the (SMOTE-augmented) minority class and it decreases its variability

SMOTE samples have the same expected value as the original minority class samples ($E(X_{j}^{\text{SMOTE}})=E(X_{j})$), but smaller variance ($\text{var}(X_{j}^{\text{SMOTE}})=\frac{2}{3}\text{var}(X_{j})$).

##### Practical consequences

The overall expected value of the SMOTE-augmented minority class is equal to the expected value of the original minority class, while its variance is smaller. Therefore, SMOTE has little impact on the classifiers that base their classification rules on class-specific mean values and overall variances (as DLDA), while it has some (harmful) impact on the classifiers that use class-specific variances (as DQDA), because they use biased estimates.

SMOTE impacts also variable selection. For example, the p-values obtained comparing two classes with a *t*-test after SMOTE-augmenting the data are smaller than those obtained using the original data (SMOTE reduces the standard error increasing the sample size and decreasing the variance, while the difference between the sample means does not change much). This can misleadingly indicate that many variables are differentially expressed between the classes. SMOTE does not substantially alter the ranking of the variables by their *t* statistics: the overlap between the variables selected using original or SMOTE-augmented data is substantial when the number of selected variables is kept fixed.

#### SMOTE introduces correlation between some samples, but not between variables

SMOTE does not introduce correlation between different variables. The SMOTE samples are strongly positively correlated with the samples from the minority class used to generate them (**x** and **x**^**R**^ from Eq. 1) and with the SMOTE samples obtained using the same original samples.

##### Practical consequences

SMOTE can be problematic for the classifiers that assume independence among samples, as for example penalized logistic regression or discriminant analysis methods. Also, performing variable selection after using SMOTE should be done with some care because most variable selection methods assume that the samples are independent.

#### SMOTE modifies the Euclidean distance between test samples and the (SMOTE-augmented) minority class

When data are high-dimensional and the similarity between samples is measured using the Euclidean distance, the test samples are on average more similar to SMOTE samples than to the original samples from the minority class.

##### Practical consequences

**Figure 1:**
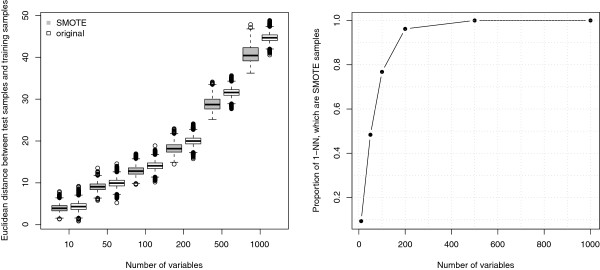
**Effect of SMOTE and the number of variables on the Euclidean distance between test samples and training set samples.** Left panel: distribution of the Euclidean distance between test and training set samples (original or SMOTE); right panel: proportion of SMOTE samples selected as nearest neighbors of test samples.

Figure 1 shows the distribution of the Euclidean distance of test samples from SMOTE and from original samples in a setting of a very moderate class-imbalance (proportion of Class 2 samples *k*_2_ = 36 / 80 = 0.45), in the null case (all variables from *N*(0, 1)). As the number of variables increases, the difference between the two distributions becomes more marked: the test samples are closer to the SMOTE samples than to the original samples. Therefore, when the number of variables is sufficiently large (*p* = 300 with these settings, right panel of Figure 1), the “nearest neighbor” of any test sample is one of the SMOTE samples, which belongs to the minority class.

This phenomenon is present also when there are some differences between classes but few variables truly discriminate the classes. This is often the case for high-dimensional data and it has important practical implications. For example, when the number of variables is large, SMOTE is likely to bias the classification towards the minority class for k-NN classifiers that measure the similarity between samples using the Euclidean distance. Conversely, SMOTE does not bias the classification towards the minority class if the number of variables is small, as the Euclidean distance of new samples from both classes is similar for the null variables (Figure 1). For these reasons SMOTE seems useful in reducing the class-imbalance problem for k-NN when the number of variables is small or if the number of variables is reduced using variable selection methods (see simulation results and the analyses of empirical data for further insights).

### Results on simulated data

Simulations were used to systematically explore the behavior of SMOTE with high-dimensional data and to show empirically the consequences of the theoretical results. Under the null case the class membership was randomly assigned, while in the alternative case the class-membership depended on some of the variables. If not stated otherwise, the results refer to simulations where the variables were correlated (*ρ* = 0.8), the samples (but not the variables) were normalized and SMOTE was used before variable selection. In the alternative case we present the results where the difference between classes was moderate (*μ*^(2)^ = 1).

#### Classification of low-dimensional data (*p* = *G* = 5, *n*_*train*_ = 40, 80, 200, *k*_1_ = 0.10)

**Figure 2:**
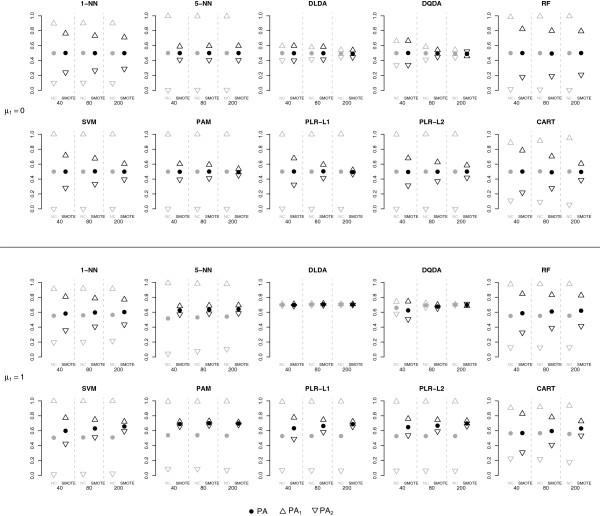
**Classification results using low-dimensional data.** Predictive accuracy (overall (PA) and class-specific (PA_1_, PA_2_)) achieved with SMOTE (black symbols) or without any class-imbalance correction (NC gray symbols) for 7 types of classifiers, for different training set sample sizes (40, 80 or 200 samples).

The (uncorrected) classifiers trained on low-dimensional class-imbalanced data assigned most of the samples to the majority class, both in null and in alternative case (Figure 2); the classifiers with the smallest bias towards the majority class were DLDA (not biased in the alternative case) and DQDA, for which the bias decreased as the sample size increased. SMOTE did not seem to impact the performance of these classifiers (only marginally for DQDA, increasing the bias in the alternative case), while it reduced the bias towards the majority class for k-NN (most notably for 5-NN), PLR-L1, PLR-L2 and PAM, performing well also when the sample size was small (*n* = 40) and increasing the overall predictive accuracy (PA) in the alternative case. A similar but attenuated effect was observed for the other classifiers (CART, SVM, RF) where SMOTE decreased the difference between class-specific PA, most notably for large sample sizes, but did not remove it. Similar results were obtained using *p* = *G* = 10 variables (data now shown).

#### Classification of high-dimensional data (*p* = 1, 000, *G* = 1, 000 or 40, *n*_*train*_ = 80)

**Figure 3:**
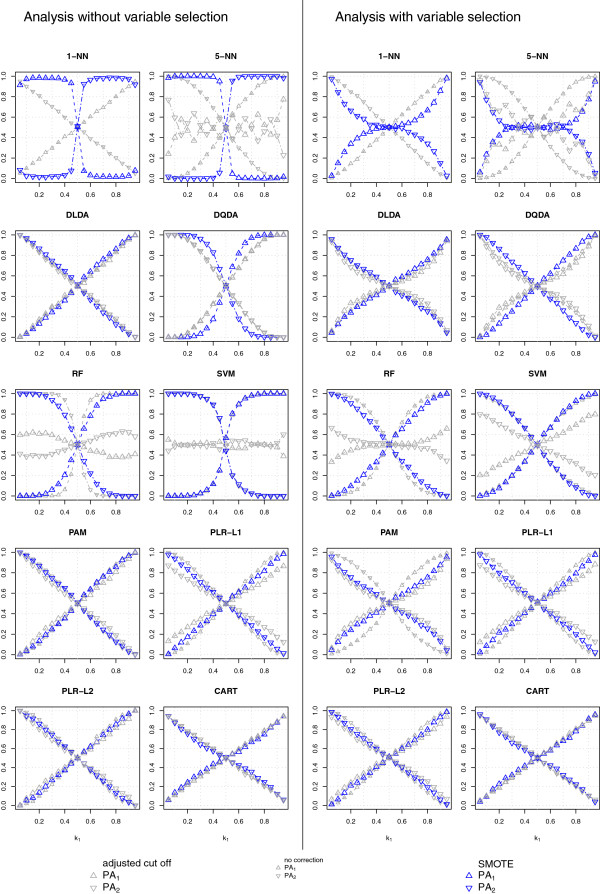
**Null case classification results for high-dimensional data.** Class-specific predictive accuracies (PA_1_, PA_2_) achieved with SMOTE (blue symbols), without any class-imbalance correction (small, gray symbols) and with cut-off adjustment (large, gray symbols) for 7 types of classifiers, varying the proportion of Class 1 samples in the training set (*k*_1_).

**Figure 4:**
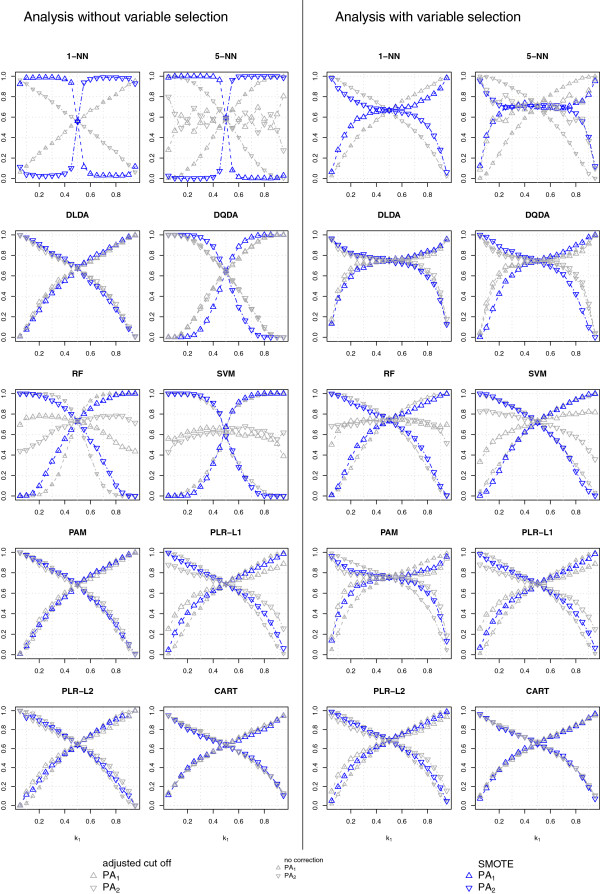
**Alternative hypothesis classification results for high-dimensional data.** Symbols as in Figure 3.

Figure 3 (null case) and Figure 4 (alternative case) display the classification results obtained using high-dimensional data. All the uncorrected classifiers assigned most of the test samples to the majority class, whether we used all variables (*G* = 1, 000) or only a selected subset (*G* = 40). The probability of classifying a new sample in the majority class increased with the level of class-imbalance for all classifiers and was larger in the null case, while variable selection decreased the bias towards the majority class for most classifiers, with the exception of k-NN. Interestingly, the discrepancy between the class-specific PA was large also for DLDA and DQDA, which were the least sensitive to the class-imbalance problem in the low-dimensional setting. These results are in line with those reported previously [7].

Adjusting the classification threshold substantially decreased the class-imbalance bias of 5-NN, RF and SVM (more effectively when variable selection was not performed), and was helpful to some extent also for PAM, provided that variable selection was performed. A slight improvement was observed also for PLR-L1 (more obvious when variable selection was not performed) and PLR-L2, while this strategy was not effective for the other classifiers. The peculiar behavior of 5-NN with classification threshold is expected, as under the null hypothesis the class specific probabilities are piecewise monotone functions of class-imbalance with breakpoints at *k*_1_ = 1 / 5, 2 / 5, 3 / 5, 4 / 5.

SMOTE had only a small impact on the class-specific PA of all the classifiers other than k-NN and PAM: SMOTE either further increased the probability of classification in the majority class (DQDA and SVM, and almost imperceptibly for DLDA) or slightly decreased it (RF, PLR-L1, PLR-L2 and CART). However, the overall effect of SMOTE was almost negligible.

SMOTE had the most dramatic effect on k-NN classifiers but the effectiveness of its use depended on the variable selection strategy. SMOTE classified most of the new samples in the minority class for any level of class-imbalance when all the variables were used, while it reduced the bias observed in the uncorrected analyses when used with variable selection: the class-specific PA of the two classes were approximately equal for a wide range of class-imbalance levels, especially for 3-NN and 5-NN, both in the null and in the alternative case.

To a lesser extent, SMOTE with variable selection was beneficial also in reducing the class-imbalance problem of PAM, decreasing the number of samples classified in the majority class, both in the null and in the alternative case; this was not the case when PAM was used without prior variable selection. A possible explanation of this behavior is given in the Additional file 2.

Similar conclusions would be obtained using AUC and G-mean to interpret the results (Additional file 3). SMOTE without variable selection reduced the G-mean for k-NN, DQDA and SVM, it increased it for RF, PLR-L1, PLR-L2 and PAM (when the class-imbalance was large) and did not change it for DLDA and CART. The AUC values were very similar using SMOTE or uncorrected analysis, but SMOTE with variable selection increased AUC and G-mean values for k-NN and PAM.

Performing variable selection before or after SMOTE did not significantly impact the performance of the classification methods (data not shown). In general, the results observed in the alternative case were similar to those observed in the null case, suggesting that our theoretical findings are relevant also in the situations where the class-membership depends on some of the variables. When the differences between the classes were larger, the class-imbalance problem was less severe, therefore using SMOTE was less helpful (data not shown).

**Figure 5:**
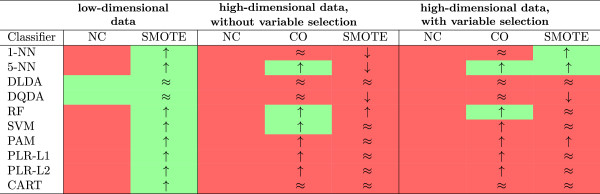
**Summary of results obtained on the simulated data.** Green and red color shading denote good and poor performance of the classifiers, respectively. Upwards and downwards trending arrows and the symbol ≈ denote improved, deteriorated or similar performance of the classifier when comparing SMOTE or adjusted classification threshold (CO) with the uncorrected analysis (NC).

Similar conclusions were obtained when all the variables were differentially expressed (Additional file 4) or were simulated from the exponential distribution (Additional file 5). See also Figure 5 for a visual summary of the results.

### Results from the experiments on gene expression data sets

**Figure 6:**
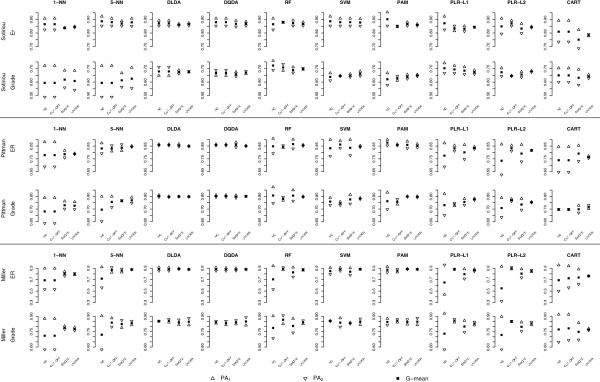
**Class-specific predictive accuracies (PA**_**1**_**, PA**_**2**_**), AUC and G-mean for experimental data.** NC: No correction, original data used; CUT-OFF: results obtained by changing the classification threshold; UNDER: simple undersampling.

We analyzed three high-dimensional gene expression data sets, performing two prediction tasks on each of them (Table 2). These experiments were performed to validate the results from the simulation study and to show the practical application of our theoretical results. Uncorrected analysis, analysis with the adjusted classification threshold (cut-off adjustment), SMOTE and simple undersampling [2] results were displayed presenting average class-specific PA and G-mean (Figure 6; more detailed results are available in Additional file 6).

**Table 2:** Experimental data sets

**Data set**	**Prediction task**	**Number of features**	**Number of samples**	***n***_***min***_	***k***_***min***_	**Minority class**
Sotiriou	ER	7,650	99	34	0.34	ER-
	Grade	7,650	99	45	0.45	Grade 3
Pittman	ER	12,625	158	48	0.30	ER-
	Grade	12,625	158	63	0.40	Grade 1 or 2
Miller	ER	22,283	247	34	0.14	ER-
	Grade	22,283	249	54	0.22	Grade 3

Number of samples, number of samples in the minority class (*n*_*min*_), level of class-imbalance (*k*_*min*_) and number of features for the analyzed gene expression data sets and different classification tasks.

The experimental results were very consistent with the simulation results. Most uncorrected classifiers seemed to be sensitive to class-imbalance, even when the class-imbalance was moderate. With few exceptions, the majority class had a better class-specific PA (most notably for k-NN, RF, PLR-L1, PLR-L2 and CART); the larger differences were seen when the class-imbalance was large (Miller’s and Pittman’s data) and for harder classification tasks (grade). The class-specific PA of DLDA and DQDA were about the same for all the classification tasks; these classifiers, together with PAM, had the largest AUC and G-mean values and seemed the least sensitive to class-imbalance. SMOTE, cut-off adjustment and undersampling had little or no effect on their classification results.

Changing the cut-off point decreased the class-imbalance bias of RF, SVM, PAM, PLR-L1 and PLR-L2 and 5-NN (with the exception of the results obtained on the Sotiriou’s data) and outperformed undersampling, while it was inefficient with the other classifiers.

SMOTE with variable selection had the most dramatic effect on k-NN classifiers, substantially reducing the discrepancy between the class-specific PA, generally increasing the G-mean and, to a lesser extent, the AUC values (Miller’s data); in this case SMOTE performed similarly, but not better, than undersampling. On the other hand, when variable selection was not performed SMOTE worsened the performance of k-NN: most samples were classified in the minority class and the AUC and G-mean values substantially decreased, while undersampling performed better than uncorrected analysis (Table 3 for results on Miller’s data and Additional file 6 for Sotiriou’s and Pittman’s data).

**Table 3:** Performance of the classifiers on the Miller data set without feature selection

		**ER**			**Grade**		
		**1-NN**	**3-NN**	**5-NN**	**1-NN**	**3-NN**	**5-NN**
**NC (CUT-OFF)**	PA	0.838	0.862	0.874 (0.777)	0.779	0.839	0.835 (0.835)
	PA_1_	0.925	0.953	0.972 (0.789)	0.897	0.954	0.949 (0.897)
	PA_2_	0.294	0.294	0.265 (0.706)	0.352	0.426	0.426 (0.611)
	AUC	0.610	0.692	0.772 (0.772)	0.625	0.769	0.816 (0.816)
	G-mean	0.522	0.529	0.507 (0.746)	0.562	0.637	0.636 (0.741)
**SMOTE**	PA	0.271	0.249	0.249	0.364	0.373	0.384
		(0.012)	(0.013)	(0.012)	(0.014)	(0.015)	(0.016)
	PA_1_	0.156	0.130	0.132	0.194	0.209	0.223
		(0.014)	(0.014)	(0.013)	(0.018)	(0.020)	(0.020)
	PA_2_	0.996	0.992	0.984	0.979	0.966	0.966
		(0.010)	(0.015)	(0.017)	(0.012)	(0.013)	(0.011)
	AUC	0.576	0.632	0.671	0.586	0.680	0.736
		(0.009)	(0.014)	(0.013)	(0.011)	(0.013)	(0.010)
	G-mean	0.393	0.359	0.360	0.435	0.449	0.464
		(0.018)	(0.020)	(0.019)	(0.020)	(0.021)	(0.021)
**UNDER**	PA	0.625	0.685	0.691	0.766	0.836	0.840
		(0.065)	(0.056)	(0.049)	(0.017)	(0.012)	(0.012)
	PA_1_	0.742	0.841	0.863	0.798	0.871	0.878
		(0.017)	(0.013)	(0.012)	(0.016)	(0.011)	(0.012)
	PA_2_	0.761	0.866	0.890	0.649	0.709	0.700
		(0.017)	(0.013)	(0.010)	(0.051)	(0.039)	(0.028)
	AUC	0.693	0.822	0.861	0.723	0.833	0.850
		(0.033)	(0.021)	(0.021)	(0.027)	(0.015)	(0.008)
	G-mean	0.689	0.770	0.784	0.719	0.786	0.784
		(0.036)	(0.031)	(0.029)	(0.029)	(0.022)	(0.017)

Overall predictive accuracy (PA), predictive accuracy for Class 1 (*PA*_1_), predictive accuracy for Class 2 (*PA*_2_), Area under the ROC curve (AUC) and G-mean for 1-NN, 3-NN and 5-NN achieved on the Miller data set with different methods of training set manipulation (no correction - NC (in brackets we report the results obtained by adjusting the threshold for 5-NN - CUT-OFF), SMOTE and undersampling - UNDER). Prediction of Estrogen receptor status (**ER**) and Grade of the tumor (**Grade**). All variables were considered when training the classifiers.

SMOTE reduced the discrepancy in class-specific PA for the other classifiers (RF, SVM, PAM, PLR-L1, PLR-L2 and CART), but simple undersampling performed very similarly (PAM) or better (RF, SVM, PLRL1, PLR-L2 and CART).

### Results obtained modifying the class-imbalance of Sotiriou’s data

**Figure 7:**
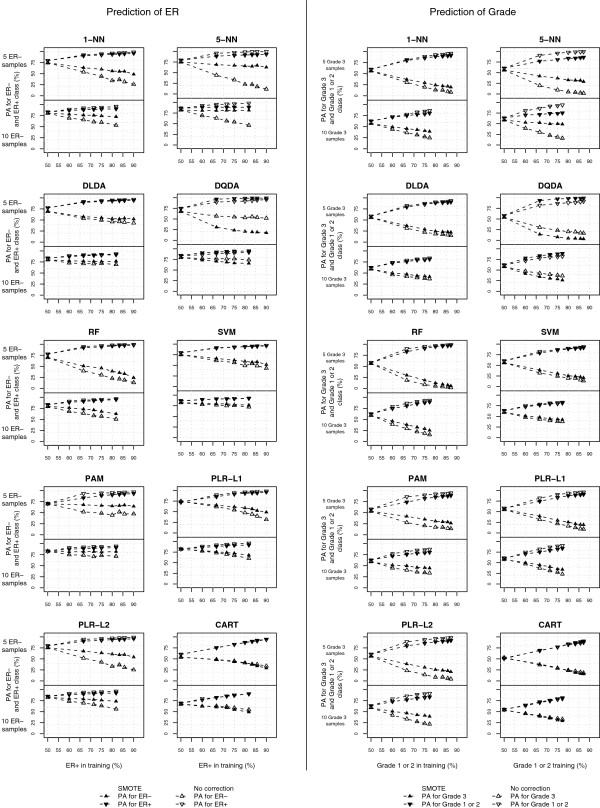
**Class-specific predictive accuracies for Sotiriou’s data, varying class imbalance.** Left panels: prediction of ER, ER- is the minority class. Right panel: prediction of grade, grade 3 is the minority class. The sample size of the minority class is fixed to *n*_*min*_ = 5 (upper panels) or *n*_*min*_ = 10 (lower panels), while it varies for the majority class.

To get a better insight into the class-imbalance problem, we obtained different levels of class-imbalance on Sotiriou’s data set and compared the performance of SMOTE with uncorrected analysis and undersampling. Figure 7 displays the average class-specific PA for ER classification (left panel) and grade (right panel); the leftmost points of each graph show the results from simple undersampling and the total sample size increases with class-imbalance.

For the uncorrected classifiers the PA of the minority class markedly decreased as the class-imbalance increased, despite of the fact that the sample size of the training set was larger. This effect was more pronounced when the differences between classes were smaller (grade classification) or for smaller sample sizes (*n*_1_ = 5).

For most classifiers SMOTE improved the PA of the minority class, compared to the uncorrected analyses. The classifiers that benefited the most from the use of SMOTE were the k-NN classifiers, especially 5-NN (note that variable selection was performed); SMOTE was somehow beneficial also for PAM, PLR-L1 and PLR-L2, while the minority class PA improved only moderately for DLDA, RF, SVM and CART, and decreased for DQDA. However, SMOTE did not remove the class-imbalance problem and, even if it was beneficial compared to the uncorrected analysis, it generally performed worse than undersampling. The exceptions were PAM and 5-NN for ER classification (but not for grade), where the drop in the PA of the minority class was very moderate. Overall, the classification results were in line with the simulation results and confirmed our theoretical findings.

## Discussion

The classifiers that we considered in this study were previously shown to be sensitive to class-imbalance: the predictive accuracy of the minority class tends to be poor and they tend to classify most test samples in the majority class, even when there are no differences between the classes. The high-dimensionality further increases the bias towards the classification in the majority class; undersampling techniques seem to be helpful in reducing the class-imbalance problem for high-dimensional data, while simple oversampling [2] is not [7].

In this article we focused on high-dimensional data and investigated the performance of SMOTE, an oversampling approach that creates synthetic samples. We explored the properties of SMOTE on high-dimensional data from a theoretical and empirical point of view, using simulation studies and breast cancer gene expression microarray data. The performance of the classifiers was evaluated with overall and class specific predictive accuracies, area under the ROC curve (AUC) and G-mean.

Most of the classifiers that we considered benefit from SMOTE if data are low-dimensional: SMOTE reduces the bias towards the classification in the majority class for k-NN, SVM, PAM, PLR-L1, PLR-L2, CART and, to some extent, for RF, while it hardly affects the discriminant analysis classifiers (DLDA and DQDA). On the other hand, for high-dimensional data SMOTE is not beneficial in most circumstances: it performs similarly to uncorrected class-imbalanced classification and worse than cut-off adjustment or simple undersampling.

In practice, only k-NN classifiers seem to benefit substantially from the use of SMOTE in the high-dimensional setting, provided that variable selection is performed before using SMOTE; the benefit is larger if more neighbors are used. SMOTE for k-NN without variable selection should not be used, because it surprisingly biases the classification towards the minority class: we showed that the reason lies in the way SMOTE modifies the Euclidean distance between the new samples and the minority class. Our theoretical proofs made many assumptions; however, analyzing the simulated and real data, where the assumptions were violated, we observed that our results were valid in practice.

We showed that for high-dimensional data SMOTE does not change the mean value of the SMOTE-augmented minority class, while it reduces its variance; the practical consequence of these results is that SMOTE hardly affects the classifiers that base their classification rules on class specific means and overall variances; such classifiers include the widely used DLDA. Additionally, SMOTE harms the classifiers that use class-specific variances (as DQDA), as it produces biased estimates: our experimental data confirmed these finding, showing that SMOTE further increased the bias towards the majority class. SMOTE should therefore not be used with these types of classifiers.

For the other classifiers it is more difficult to isolate the reasons why SMOTE might or might not work on high-dimensional data. SMOTE has a very limited impact on SVM and CART. PLR-L1, PLR-L2 and RF seem to benefit from SMOTE in some circumstances, however the improvements in the predictive accuracy of the minority class seem moderate when compared to the results obtained using the original data and can be probably attributed to the balancing of the training set. The apparent benefit of SMOTE for PAM is limited to situations where variable selection is performed before using PAM, which is not a normally used procedure, and can be explained as the effect of removing the PAM-embedded class-imbalance correction, which increases the probability of classifying a sample in the majority class.

Using the gene expression data we compared SMOTE with simple undersampling, the method that obtains a balanced training set by removing some of the samples from the majority class. Our results show that for RF, SVM, PLR, CART and DQDA simple undersampling seems to be more useful than SMOTE in improving the predictive accuracy of the minority class without largely decreasing the predictive accuracy of the majority class. SMOTE and simple undersampling perform similarly for PAM (with variable selection) and DLDA; similar results were obtained by others also for low-dimensional data [8]. Sometimes SMOTE performs better than simple undersampling for k-NN (with variable selection). Our results are in agreement with the finding that SMOTE had little or no effect on SVM when data were high-dimensional [19].

The results showing that simple undersampling ourperforms SMOTE might seem surprising, as this method uses only a small subset of the data. In practice undersampling is effective in removing the gap between the class-specific predictive accuracies for high-dimensional data [7] and it is often used as a reasonable baseline for algorithmic comparison [35]. One of its shortcomings is the large variability of its estimates, which can be reduced by bagging techniques that use multiple undersampled training sets. We previously observed that bagged undersampling techniques outperform simple undersampling for high-dimensional data, especially when the class-imbalance is extreme [7]. Others showed that bagged undersampling techniques outperformed SMOTE for SVM with high-dimensional data [19]. Therefore, we expect that the classification results presented in this paper could be further improved by the use of bagged undersampling methods.

We devoted a lot of attention to studying the performance of SMOTE in the situation where there was no difference between the classes or where most of the variables did not differ between classes. We believe that in this context these situations are extremely relevant. It is well known that most of the problems arising from learning on class-imbalanced data arise in the region where the two class-specific densities overlap. When the difference between the class-specific densities is large enough, the class-imbalance does not cause biased classification for the classifiers that we considered, even in the high-dimensional setting [7]. The other reason is that when a very large number of variables is measured for each subject, in most situations the vast majority of variables do not differentiate the classes and the signal-to-noise ratio can be extreme. For example, Sotiriou et al. [36] identified 606 out of the 7,650 measured genes as discriminating ER+ from ER- samples in their gene expression study; at the same time ER status was the known clinico-pathological breast cancer phenotype for which the largest number of variables was identified (137 out of the 7,650 genes discriminated grade, 11 out of the 7,650 node positivity, 3 out of the 7,650 tumor size and 13 out of the 7,650 menopausal status). Similar results can be found in most gene expression microarray studies, where rarely more than few hundreds of genes differentiate the classes of interest. Furthermore, the results from the simulation studies where all the variables were differentially expressed were consistent with those obtained when only few variables differentiated the classes, indicating that our conclusions are not limited to sparse high-dimensional data.

Variable selection is generally advisable for high-dimensional data, as it removes some of the noise from the data [37]. SMOTE does not affect the ranking of variables if the variable selection method is based on class-specific means and variances. For example, when variable selection is based on a two-sample *t*-test and a fixed number of variables are selected, as in our simulations, the same results are obtained if variable selection is performed before or after using SMOTE. However, the results obtained by performing variable selection on SMOTE-augmented data must be interpreted with great care. For example, the p-values of a two-sample *t*-test are underestimated and should not be interpreted other than for ranking purposes: if the number of variables to select depends on a threshold on the p-values it will appear that many variables are significantly different between the classes. Another reason of concern is that SMOTE introduces some correlation between the samples and most variable selection methods (as well as some classifiers) assume the independence among samples.

Many variants of the original version of SMOTE exist, however in this paper we only considered the original version of SMOTE. The variants of SMOTE are very similar in terms of the expected value and variance of the SMOTE samples, as well as the expected value and variance of the Euclidean distance between new samples and samples from the SMOTE-augmented data set. Under the null hypothesis all the theoretical results presented in this paper would apply also for Borderline-SMOTE [22] and Safe-Level-SMOTE [23]. Further research would be needed to assess the performance of these algorithms for high-dimensional data when there is some difference between the classes.

We considered only a limited number of simple classification methods, which are known to perform well in the high-dimensional setting, where the use of simple classifiers is generally recommended [37]. Our theoretical and empirical results suggest that many different types of classifiers do not benefit from SMOTE if data are high-dimensional; the only exception that we identified are the k-NN classifiers. It is however possible that also in the high-dimensional setting SMOTE might be more beneficial for some classifiers that were not included in our study.

## Conclusions

SMOTE is a very popular method for generating synthetic samples that can potentially diminish the class-imbalance problem. We applied SMOTE to high-dimensional class-imbalanced data (both simulated and real) and used also some theoretical results to explain the behavior of SMOTE. The main findings of our analysis are: 

• in the low-dimensional setting SMOTE is efficient in reducing the class-imbalance problem for most classifiers;

• SMOTE has hardly any effect on most classifiers trained on high-dimensional data;

• when data are high-dimensional SMOTE is beneficial for k-NN classifiers if variable selection is performed before SMOTE;

• SMOTE is not beneficial for discriminant analysis classifiers even in the low-dimensional setting;

• undersampling or, for some classifiers, cut-off adjustment are preferable to SMOTE for high-dimensional class-prediction tasks.

Even though SMOTE performs well on low-dimensional data it is not effective in the high-dimensional setting for the classifiers considered in this paper, especially in the situations where signal-to-noise ratio in the data is small.

## Methods

### Notation

Let *x*_*ij*_ be the value of *j*th variable (*j* = 1, ..., *p*) for the *i*th sample (*i* = 1, ..., *n*) that belongs to Class *c* (*c* = 1 or 2), *k*_*c*_ = *n*_*c*_ / *n* is the proportion of samples from Class *c* and *n*_*c*_ is the number of samples in class *c*. Let the sample size of the minority class be denoted by *n*_*min*_. Let us say we limit our attention to *G* ≤ *p* variables that are the most informative about the class distinction. Capital letters (as *X*) denote random variables while lowercase letters (as *x*) denote observations; bold letters (**x**) indicate set of variables. The Gaussian distribution with mean *μ* and standard deviation *σ* is indicated with *N*(*μ*, *σ*) and the uniform distribution defined on [0,1] with *U*(0, 1).

### SMOTE

SMOTE [9] is an oversampling technique that generates synthetic samples from the minority class using the information available in the data. For each sample from the minority class (**x**) 5 (or *n*_*min*_ - 1 if *n*_*min*_ ≤ 5) samples from the minority class with the smallest Euclidean distance from the original sample were identified (nearest neighbors), and one of them was randomly chosen (**x**^*R*^). The new synthetic SMOTE sample was defined as

(2) $$\text{S}=\text{x}+u\cdot ({\text{x}}^{R}-\text{x}),$$

where *u* was randomly chosen from *U*(0, 1). *u* was the same for all variables, but differed for each SMOTE sample; this choice guarantees that the SMOTE sample lies on the line joining the two original samples used to generate it [2,9]. By SMOTE-augmenting the minority class we obtained a class-balanced training set, as suggested in [8].

### Simple undersampling

Simple undersampling (down-sizing) consists of obtaining a class-balanced training set by removing a subset of randomly selected samples from the larger class [2]. The undersampled training set can be considerably smaller than the original training set if the class-imbalance is large. Simple undersampling was used only for the analysis of the experimental data sets.

### Cut-off adjustment

We attempted to adjust for the class-imbalance by changing the classification threshold of the classifiers. For each classifier we estimated the posterior probability of classification in Class 1 for the new samples ($\hat{p}(c=1|x^{*})$). The classification rule was then defined as: classify at random if *p*(*c* = 1|**x**^∗^) = *k*_1_, classify to Class 1 when $\hat{p}(c=1|x^{*})>k_{1}$ and to Class 2 otherwise. (Note that the uncorrected classifiers use the threshold value of 0.5 for any level of class imbalance.)

### Data simulation of high-dimensional data

We simulated *p* = 1, 000 variables for each of *n* = 100 samples. The variables were simulated under a block exchangeable correlation structure, in which the 10 variables within each block had a pairwise correlation of *ρ* = 0.8, 0.5, 0.2 or 0 (independence case), while the variables from different blocks were independent [38]. The data set was split into a training set (*n*_*train*_ = 80) and a balanced test set (*n*_*test*_ = 20). Different levels of class-imbalance were considered for the training sets, varying the proportion of samples from Class 1 from *k*_1_ = 0.05 to 0.95.

Under the null case the class membership was randomly assigned and all the variables were simulated from *N*(0, 1). Under the alternative case, the class membership was dependent on the values of *p*_*DE*_ = 20 non-null variables, generated from *N*(0, 1) in Class 1 and from *N*(*μ*^(2)^, 1) in Class 2 (*μ*^(2)^ = 0.5, 0.7, 1, 2); the remaining variables were simulated as in the null case. We considered also a situation where all variables were differentially expressed. In this setting we used *μ*^(2)^ = 0.2, which assured a similar predictive power as in the situation where we used sparse data and moderate differences between the classes (*p*_*DE*_ = 20 and *μ*^(2)^ = 1).

We performed also a limited set of simulations where all the variables were simulated from the exponential distribution with rate equal to one. In the alternative case a number randomly generated from *U*(1, 1.5) was added to the *p*_*DE*_ = 20 non-null variables in Class 2.

Each simulation was repeated 1,000 times and overall more than 11 million classifiers were trained.

### Data simulation of low-dimensional data

We performed also a limited number of simulations where data were low-dimensional. We simulated and used *p* = *G* = 5 or 10 variables and varied the size of the training set (*n*_*train*_ = 40, 80 and 200), keeping the level of class-imbalance fixed (*k*_1_ = 0.10). The test sets were balanced (*n*_*test*_ = 40). All the variables were correlated (*ρ* = 0.8) and simulated as described for the high-dimensional data (*μ*^(2)^ = 1 for the alternative case).

### Data normalization, variable selection and derivation of the classifiers

We evaluated the effect of data normalization, developing classification rules (i) using raw data (*x*_*ij*_), (ii) normalizing the samples ($x_{\text{ij}}^{s}=x_{\text{ij}}-\frac{1}{p}\overset{p}{\underset{k=1}{\sum }}x_{\text{ik}}$) and (iii) normalizing the variables ($x_{\text{ij}}^{v}=x_{\text{ij}}-\frac{1}{n}\overset{n}{\underset{k=1}{\sum }}x_{\text{kj}})$. Normalization was performed separately on the training and test set, before variable selection or augmentation of the training set. Data normalizatoin was not performed when all the variables were differentially expressed.

We used all the variables (*p* = *G*) or selected *G* = 40 variables with the largest absolute t-statistics derived from the two sample *t*-test with assumed equal variances; variable selection was performed on the training set, either before or after using SMOTE but only after using undersampling (this strategy outperforms variable selection before undersampling [7]).

The classification rules were derived completely on the training set, using seven types of classification methods: k-NN with *k* = 1, 3 or 5, discriminant analysis (DLDA and DQDA), RF, SVM, PAM, penalized logistic regression (PLR) with linear penalty (PLR-L1) and quadratic penalty (PLR-L2) and CART. For CART we used pruning, the maximum depth of any node of the final tree was set to 5 and the complexity parameter was 0.01. We used the penalized package to fit PLR; the penalization coefficient was optimized based on cross-validated likelihood. The parameters used for the other classifiers were the same as in [7], where the classifiers are shortly described.

### Evaluation of the performance of the classifiers

The classifiers were evaluated on the independent test sets, using five performance measures: (i) overall predictive accuracy (PA, the number of correctly classified samples from the test set divided by the total number of samples in the test set), (ii) predictive accuracy of Class 1 (*PA*_1_), (iii) predictive accuracy of Class 2 (*PA*_2_), (iv) Area Under the Receiver-Characteristic-Operating Curve (AUC [39]) and (v) G-mean ($\sqrt{PA_{1}\cdot PA_{2}}$). We used the function *sommers2* in the Hmisc package to compute the AUC.

### Experimental data sets

We considered three breast cancer gene expression data sets [36,40,41] and two classification tasks for each of them: prediction of estrogen receptor status (ER+ or ER-) and prediction of grade of tumors (grade 1 and 2 or grade 3). Data were pre-processed as described in the original publications. The number of variables varied from 7,650 to 22,283, the number of samples from 99 to 249, and the proportion of minority class samples from 0.14 to 0.45 (Table 2).

The classifiers were trained with *G*=40 variables, using SMOTE, simple undersampling, the uncorrected classifiers or adjusted classification threshold. Their performance was assessed with leave-one-out cross validation. To take the sampling variability into account, each classifier was trained using 50 different SMOTE-augmented or undersampled training sets. Overall, 10,878 classifiers were trained, and their performance was assessed training about one million classifiers on cross-validated training sets.

Additionally, to isolate the effect of class-imbalance, we used the Sotiriou data and obtained different levels of class-imbalance in the training set by including a randomly chosen subset of the samples in the analyses. The training sets contained a fixed number of samples in the minority class (5 or 10 ER- or grade 3 samples), while the number of samples of the majority class varied; the class-imbalance of the training sets ranged from *k*_1_=0.50 to 0.90 at most, while the test sets were class-balanced. The analysis was replicated 500 times for each level of class-imbalance, randomly selecting the samples to include in the training and test set and using SMOTE or no correction; *G*=40 variables were selected at each iteration. The results were presented as average overall and class-specific PA.

### Analysis

Analyses and simulations were carried out using R 2.8.1 [42].

## Abbreviations

SMOTE: Synthetic minority oversampling technique; CART: Classification and regression trees; PA: Predictive accuracy; PA1: Predictive accuracy for Class 1; PA2: Predictive accuracy for Class 2; k-NN: Nearest neighbor classifier with k neighbors; DLDA: Diagonal linear discriminant analysis; DQDA: Diagonal quadratic discriminant analysis; RF: Random forests; SVM: Support vector machines; PAM: Prediction analysis of microarrays; PLR: Penalized logistic regression; LOOCV: Leave-one-out cross-validation; ER: Estrogen receptor; ER+: Positive estrogen receptor; ER-: Negative estrogen receptor.

## Competing interests

The authors declare that they have no competing interests.

## Authors’ contributions

RB performed the computations and wrote the manuscript; LL designed research and wrote the manuscript. Both authors read and approved the final manuscript.

## Supplementary Material

Additional file 1 Derivation of the theoretical properties of SMOTE. Click here for file

Additional file 2 **Effect of variable selection on PAM in combination with SMOTE.** In the additional file we provide a possible explanation of the effect of variable selection on PAM used with SMOTE. Click here for file

Additional file 3 **Additional tables for the results obtained on simulated data.** In the additional file we report the AUC and G-mean obtained on simulated data. Click here for file

Additional file 4 **Results obtained on the data where all variables were differentially expressed.** The additional file reports the same information as Figure 3; all variables where differentially expressed (*p* = *p*_*DE*_ = 1, 000). Click here for file

Additional file 5 **Results obtained on the data where the variables were simulated from the exponential distribution.** The additional file reports the same information as Figure 3 for the setting where variables were simulated from the exponential distribution (page 1 - null case, page 2 - alternative case). Click here for file

Additional file 6 **Results obtained on real gene expression data sets.** The additional file reports the numerical results obtained by analyzing various gene expression data sets. Click here for file

## References

[B1] Bishop CM Pattern Recognition and Machine Learning (Information Science and Statistics) 2007 New York: Springer

[B2] He H Garcia EA Learning from imbalanced data IEEE Trans Knowledge Data Eng 2009 21 9 1263 1284

[B3] Daskalaki S Kopanas I Avouris N Evaluation of classifiers for an uneven class distribution problem Appl Artif Intell 2006 20 5 381 417 10.1080/08839510500313653

[B4] Ramaswamy S Ross KN Lander ES Golub TR A molecular signature of metastasis in primary solid tumors Nat Genet 2003 33 49 54 10.1038/ng1060 12469122

[B5] Shipp MA Ross KN Tamayo P Weng AP Kutok JL Aguiar RC Gaasenbeek M Angelo M Reich M Pinkus GS Ray TS Koval MA Last KW Norton A Lister TA Mesirov J Neuberg DS Lander ES Aster JC Diffuse large B-cell lymphoma outcome prediction by gene-expression profiling and supervised machine learning Nat Med 2002 8 68 10.1038/nm0102-68 11786909

[B6] Iizuka N Oka M Yamada-Okabe H Nishida M Maeda Y Mori N Takao T Tamesa T Tangoku A Tabuchi H Hamada K Nakayama H Ishitsuka H Miyamoto T Hirabayashi A Uchimura S Hamamoto Y Oligonucleotide microarray for prediction of early intrahepatic recurrence of hepatocellular carcinoma after curative resection Lancet 2003 361 9361 923 929 10.1016/S0140-6736(03)12775-4 12648972

[B7] Blagus R Lusa L Class prediction for high-dimensional class-imbalanced data BMC Bioinformatics 2010 11 523+ 10.1186/1471-2105-11-523 20961420 PMC3098087

[B8] Hulse JV Khoshgoftaar TM Napolitano A Experimental perspectives on learning from imbalanced data Proceedings of the 24th international conference on Machine learning 2007 Corvallis, Oregon: Oregon State University 935 942

[B9] Chawla NV Bowyer KW Hall LO Kegelmeyer WP SMOTE: synthetic minority over-sampling technique J Artif Intell Res 2002 16 341 378

[B10] Cieslak DA Chawla NW Striegel A Combating imbalance in network intrusion datasets Proc IEEE Int Conf Granular Comput 2006 Atlanta, Georgia, USA 732 737

[B11] Liu Y Chawla NV Harper MP Shriberg E Stolcke A A study in machine learning from imbalanced data for sentence boundary detection in speech Comput Speech Lang 2006 20 4 468 494 10.1016/j.csl.2005.06.002

[B12] Johnson R Chawla N Hellmann J Species distribution modelling and prediction: A class imbalance problem Conference on Intelligent Data Understanding (CIDU) 2012 9 16 10.1109/CIDU.2012.6382186

[B13] Fallahi A Jafari S An Expert System for Detection of Breast Cancer Using Data Preprocessing and Bayesian Network Int J Adv Sci Technol 2011 34 65 70

[B14] Batuwita R Palade V microPred: effective classification of pre-miRNAs for human miRNA gene prediction Bioinformatics 2009 25 8 989 995 10.1093/bioinformatics/btp107 19233894

[B15] Xiao J Tang X Li Y Fang Z Ma D He Y Li M Identification of microRNA precursors based on random forest with network-level representation method of stem-loop structure BMC Bioinformatics 2011 12 165+ 10.1186/1471-2105-12-165 21575268 PMC3118167

[B16] MacIsaac KD Gordon DB Nekludova L Odom DT Schreiber J Gifford DK Young RA Fraenkel E A hypothesis-based approach for identifying the binding specificity of regulatory proteins from chromatin immunoprecipitation data Bioinformatics 2006 22 4 423 429 10.1093/bioinformatics/bti815 16332710

[B17] Wang J Xu M Wang H Zhang J Classification of imbalanced data by using the SMOTE algorithm and locally linear embedding. International Conference on Signal Processing 2006 Guilin, China

[B18] Doyle S Monaco J Feldman M Tomaszewski J Madabhushi A An active learning based classification strategy for the minority class problem application to histopathology annotation BMC Bioinformatics 2011 12 424+ 10.1186/1471-2105-12-424 22034914 PMC3284114

[B19] Wallace B Small K Brodley C Trikalinos T Class imbalance, Redux Data Mining (ICDM), 2011 IEEE 11th International Conference on 2011 Vancouver, Canada 754 763

[B20] Ertekin SE Huang J Bottou L Giles CL Learning on the border: Active learning in imbalanced data classification Proceedings of ACM Conference on Information and Knowledge Management 2007 Lisbon, Portugal 127 136

[B21] Radivojac P Chawla NV Dunker AK Obradovic Z Classification and knowledge discovery in protein databases J Biomed Inform 2004 37 4 224 239 10.1016/j.jbi.2004.07.008 15465476

[B22] Han H Wang WY Mao BH Borderline-SMOTE: A New Over-Sampling Method in Imbalanced Data Sets Learning Advances in Intelligent Computing Volume 3644 of Lecture Notes in Computer Science 2005 Berlin/Heidelberg: Springer 878 887

[B23] Bunkhumpornpat C Sinapiromsaran K Lursinsap C Safe-Level-SMOTE:Safe-Level-Synthetic Minority Over-Sampling TEchnique for Handling the Class Imbalanced Problem Advances in Knowledge Discovery and Data Mining, Volume 5476 2009 Berlin / Heidelberg: Springer 475 482

[B24] Gu Q Cai Z Zhu L Classification of Imbalanced Data Sets by Using the Hybrid Re-sampling Algorithm Based on Isomap Advances in Computation and Intelligence Volume 5821 of Lecture Notes in Computer Science 2009 Berlin / Heidelberg: Springer 287 296

[B25] Breiman L Friedman JH Olshen RA Stone CJ Classification and Regression Trees 1984 Boca Raton: Chapman & Hall/CRC

[B26] Fix E Hodges JJL Discriminatory analysis. Nonparametric discrimination: consistency properties Int Stat Rev 1989 57 3 238 247 10.2307/1403797

[B27] Speed TP Statistical Analysis of Gene Expression Microarray Data 2003 Boca Raton: Chapman & Hall/CRC

[B28] Simon RM Korn EL McShane LM Radmacher MD Wright GW Zhao Y Design and Analysis of DNA Microarray Investigations 2004 New York: Springer

[B29] Breiman L Random forests Mach Learn 2001 45 5 32 10.1023/A:1010933404324

[B30] Cortes C Vapnik V Support-vector networks Mach Learn 1995 20 3 273 297

[B31] Tibshirani R Hastie T Narasimhan B Chu G Diagnosis of multiple cancer types by shrunken centroids of gene expression Proc Natl Acad Sci USA 2002 99 10 6567 6572 10.1073/pnas.082099299 12011421 PMC124443

[B32] Zhu J Hastie T Classification of gene microarrays by penalized logistic regression Biostatistics 2004 5 3 427 443 10.1093/biostatistics/kxg046 15208204

[B33] Beyer K Goldstein J Ramakrishnan R Shaft U When is “nearest neighbor” meaningful? Int. Conf. on Database Theory 1999 Jerusalem, Israel 217 235

[B34] Hinneburg A Aggarwal CC Keim DA What is the nearest neighbor in high dimensional spaces? Proc 26th Int Conf Very Large Data Bases, VLDB ’00 2000 San Francisco 506 515

[B35] Drummond C Holte RC C4.5, Class Imbalance, and Cost Sensitivity: Why Under-Sampling beats Over-Sampling Workshop on Learning from Imbalanced Datasets II, ICML 2003 Ottawa, Canada

[B36] Sotiriou C Neo SY McShane LM Korn EL Long PM Jazaeri A Martiat P Fox SB Harris AL Liu ET Breast cancer classification and prognosis based on gene expression profiles from a population-based study Proc Natl Acad Sci USA 2003 100 18 10393 10398 10.1073/pnas.1732912100 12917485 PMC193572

[B37] Dudoit S Fridlyand J Speed TP Comparison of discrimination methods for the classification of tumors using gene expression data J Am Stat Assoc 2002 97 457 77 87 10.1198/016214502753479248

[B38] Guo Y Hastie T Tibshirani R Regularized linear discriminant analysis and its application in microarrays Biostatistics 2007 8 86 100 10.1093/biostatistics/kxj035 16603682

[B39] Fawcett T An introduction to ROC analysis Pattern Recognit Lett 2006 27 8 861 874 10.1016/j.patrec.2005.10.010

[B40] Pittman J Huang E Dressman H Horng C Cheng S Tsou M Chen C Bild A Iversen E Huang A Nevins J West M Integrated modeling of clinical and gene expression information for personalized prediction of disease outcomes Proc Natl Acad Sci USA 2004 101 22 8431 8436 10.1073/pnas.0401736101 15152076 PMC420411

[B41] Miller LD Smeds J George J Vega VB Vergara L Ploner A Pawitan Y Hall P Klaar S An expression signature for p53 status in human breast cancer predicts mutation status, transcriptional effects, and patient survival Proc Natl Acad Sci USA 2005 102 38 13550 13555 10.1073/pnas.0506230102 16141321 PMC1197273

[B42] R Development Core Team R: A Language and Environment for Statistical Computing 2008 Vienna: R Foundation for Statistical Computing

