# Supplementary material for: SMOTE for high-dimensional class-imbalanced data
Source: BMC Bioinformatics. 2013 Mar 22;14:106. doi: 10.1186/1471-2105-14-106 (PMC3648438; doi:10.1186/1471-2105-14-106)
Supplement: Additional file 1 — Derivation of the theoretical properties of SMOTE. [file 1471-2105-14-106-S1.pdf]

Derivation of the theoretical properties of SMOTE

A Notation

Let $\mathbf{X} = \{X_1, X_2, \dots, X_p\}$ be the p random variables measured for a sample. Let us define the jth variable of a SMOTE sample ($\mathbf{S} = \{S_1, \dots, S_p\}$), as

$$S_j = X_j + U(R_j - X_j), \quad (1)$$

where $\mathbf{X}$ is a sample from the minority class and $\mathbf{R} = \{R_1, \dots, R_p\}$ is a randomly chosen sample among the five samples from the minority class with the smallest Euclidean distance from sample $\mathbf{X}$; U is a uniform random variable defined on the interval (0,1), independent of the other variables. The subscripts indicate the variables of a sample while the superscripts indicate the samples.

B A sample and its nearest neighbor are (almost) independent when data are high-dimensional

We performed a limited set of simulations to evaluate the distribution properties of the nearest neighbor sample ($\mathbf{X}^{\mathbf{NN}}$), the randomly chosen sample among the five nearest neighbors ($\mathbf{R}$) and their correlation with the original samples $\mathbf{X}$; we also empirically evaluated the expected value and the variance of the SMOTE samples ($\mathbf{S}$). We focused on the effect of the high-dimensionality of data, keeping the sample size fixed at 100 and varying the number of variables (from 2 to 10,000). The variables were simulated independently from three distributions (uniform, normal and exponential) with the same mean and variance (both equal to 1). The exponential distribution is positively asymmetric while the normal and uniform distributions are symmetric.

Our results suggest that when the distribution is symmetric the expected value of the nearest neighbor is the same as the expected value of the original samples, regardless of the number of variables (Table 1). When the background distribution is asymmetric the expected value of the nearest neighbor approaches the expected value of the original samples when the number of variables increases. In a similar manner, the variance of the nearest neighbor becomes similar to the variance of the original samples when the number

Table 1: Distributional properties of $\mathbf{X^{NN}}$, $\mathbf{R}$ and $\mathbf{S}$ for various distributions and number of variables. $\rho(\mathbf{S},\mathbf{X})$ denotes the correlation coefficient between SMOTE sample and the original sample used to generate it.

	Normal					Exponential					Uniform				
p	2	10	100	1,000	10,000	2	10	100	1,000	10,000	2	10	100	1,000	10,000
$E(\mathbf{X^{NN}})$	1.01	1.00	1.00	1.00	1.00	0.88	0.90	0.90	0.96	0.99	1.01	0.99	1.00	1.00	1.00
$var(\mathbf{X^{NN}})$	0.71	0.7	0.83	0.94	0.98	0.59	0.69	0.67	0.84	0.94	0.88	0.83	0.93	0.97	0.99
$\rho(\mathbf{X^{NN}},\mathbf{X})$	0.69	0.67	0.22	0.07	0.02	0.68	0.73	0.22	0.05	0.02	0.69	0.70	0.23	0.07	0.02
$E(\mathbf{R})$	1.04	1.00	1.00	1.00	1.00	0.79	0.87	0.91	0.97	0.99	1.02	0.99	1.00	1.00	1.00
$Var(\mathbf{R})$	0.62	0.70	0.86	0.95	0.98	0.45	0.62	0.69	0.87	0.95	0.82	0.86	0.94	0.98	0.99
$\rho(\mathbf{R},\mathbf{X})$	0.36	0.56	0.17	0.05	0.02	0.28	0.60	0.16	0.04	0.01	0.34	0.59	0.19	0.06	0.02
$E(\mathbf{S})$	1.03	1.00	1.00	1.00	1.00	0.90	0.93	0.95	0.98	0.99	1.01	0.99	1.00	1.00	1.00
$var(\mathbf{S})$	0.67	0.73	0.68	0.66	0.66	0.58	0.70	0.60	0.64	0.66	0.79	0.80	0.70	0.68	0.67
$\rho(\mathbf{S},\mathbf{X})$	0.74	0.86	0.71	0.66	0.63	0.67	0.87	0.73	0.65	0.63	0.69	0.87	0.70	0.66	0.64

of variables is large. Even more interestingly, the correlation between the original sample and its nearest neighbor goes to zero when the number of variables is large, meaning that the original sample and its nearest neighbor are independent when the number of variables is large. This property has already been observed by others (Beyer *et al.*, 1999; Hinneburg *et al.*, 2000).

In practice the SMOTE samples are not necessarily defined using the nearest neighbor sample, but randomly choosing one of the five nearest neighbors. This choice further reduces the distributional differences between the the original and SMOTE samples and their correlation (data not shown). In the proofs we will assume that the effectively employed nearest neighbors and the original samples have the same expected values for symmetric distributions, and that they are independent and share the same distribution for high-dimensional data.

C SMOTE does not change the expected value in the (SMOTE-augmented) minority class for high-dimensional data

Assuming that the expected value of the jth variable is the same for all the samples of the minority class ($E(X_j^s) = E(X_j)$ for samples s from the minority class)

$$E(S_j) = \frac{1}{2}\left(E(R_j) + E(X_j)\right).$$

Further assuming that the expected values of $\mathbf{R}$ and of the minority class samples are equal we obtain that

$$E(S_j) = E(X_j),$$

i.e., the expected value of the jth variable generated for the SMOTE sample is equal to the expected value of the variable from the original samples of the minority class. In practice the additional assumption is met for symmetric distributions or for high-dimensional data (Table 1).

Proof. Using the definition of eq. 1, we have

$$\begin{aligned} E(S_j) &= E\left(X_j + U(R_j - X_j)\right) \\ &= E(X_j) + E(U)\left(E(R_j) - E(X_j)\right) \\ &= \frac{1}{2}\left(E(R_j) + E(X_j)\right), \end{aligned} \tag{2}$$

because U is independent of the variables X_j and R_j, and because we assumed the equality of the expected values in the minority class. The expression further simplifies to, $E(S_j) = E(X_j)$ for symmetric or high-dimensional data (assuming that $E(R_j) = E(X_j)$). Our simulation results confirmed that in practice for symmetric distributions and for high-dimensional data the expected value of the SMOTE samples is equal to the expected value of the original samples (Table 1). Note that only the equality of the first moment is required, while it is not necessary that the variables have the same distribution in the minority class or that they are independent.

Using the same assumptions used in the previous proof, it is straightforward to show that, $E(\mathbf{S}) = \frac{1}{2}\left(E(\mathbf{R}) + E(\mathbf{X})\right)$ or, assuming that the first moments of $\mathbf{R}$ are equal to those from the minority class, $E(\mathbf{R}) = E(\mathbf{X})$.

D SMOTE decreases the variability of the (SMOTE-augmented) minority class

Assuming that the samples from the minority class are independent and have the same first two moments for the variable X_j ($E(X_j^s) = E(X_j)$ and $var(X_j^s) = var(X_j)$ for all samples s of the minority class),

$$var(S_j) = \frac{1}{3}var(X_j) + \frac{1}{3}var(R_j) + \frac{1}{3}cov(X_j, R_j)$$

$$+ \quad \frac{1}{12}E(X_j)^2 + \frac{1}{12}E(R_j)^2 - \frac{2}{12}E(X_j)E(R_j). \tag{3}$$

The expression simplifies to

$$var(S_j) = \frac{1}{3}var(X_j) + \frac{1}{3}var(R_j) + \frac{1}{3}cov(X_j, R_j) \tag{4}$$

for symmetric distributions and to

$$var(R_j) = \frac{2}{3}var(X_j). \tag{5}$$

for high-dimensional data.

Proof. The variance of a variable can be expressed as

$$var\left(S_j\right) = E\left(S_j^2\right) - E\left(S_j\right)^2. \tag{6}$$

We expand the first term of the equation

$$\begin{aligned} E\left(S_j^2\right) &= E\left(\left(X_j + U(R_j - X_j)\right)^2\right) \\ &= E\left(X_j^2 + 2U \cdot X_j\left(R_j - X_j\right) + U^2\left(R_j^2 - 2R_jX_j + X_j^2\right)\right) \\ &= \frac{1}{3}E\left(X_j^2\right) + \frac{1}{3}E\left(R_j^2\right) + \frac{1}{3}E\left(R_jX_j\right) \end{aligned} \tag{7}$$

Here we used the assumptions that the samples in the minority class are independent and that their first two moments are equal. Recall that $E(U) = 1/2$ and $E(U^2) = 1/3$ for U(0,1).

Going back to Equation 6

$$\begin{aligned} var(S_j) &= E\left(S_j^2\right) - E\left(S_j\right)^2 \\ &= \frac{1}{3}E(X_j^2) + \frac{1}{3}E\left(R_j^2\right) + \frac{1}{3}E(X_jR_j) - \left(\frac{1}{2}\left(E\left(R_j\right) + E\left(X_j\right)\right)\right)^2 \\ &= \frac{1}{3}var(X_j) + \frac{1}{3}var(R_j) + \frac{1}{3}cov(X_j, R_j) \\ &+ \frac{1}{12}E(X_j)^2 + \frac{1}{12}E(R_j)^2 - \frac{2}{12}E(X_j)E(R_j), \end{aligned} \tag{8}$$

we complete the proof.

The expression can be further simplified for symmetric distributions ($E(R_j) = E(X_j)$) and for high-dimensional data ($var(R_j) = var(X_j)$ and $cov(X_j, R_j) = 0$). Our simulation results confirmed that in practice the variance of the SMOTE samples is smaller compared to the variance of the original samples, and it is equal to two thirds of the variance of the original samples when data are high-dimensional (Figure 1).

We are interested also in the covariance between different variables for SMOTE samples ($cov(S_j, S_i)$, with $j \neq i$). Assuming that the variables are independent in the original samples ($cov(X_j, X_i) = 0$), the variables remain independent also for the SMOTE samples if the distribution is symmetric or data are high-dimensional

$$cov(S_j, S_i) = 0, j \neq i.$$

Proof. The covariance between different variables (S_j and S_i, with $i \neq j$) for a SMOTE sample is

$$\begin{aligned} cov\,(S_j, S_i) &= cov\,(X_j + U(R_j - X_j), X_i + U(R_i - X_i)) \\ &= var(U)\,(E(X_j) - E(R_j))^2 = \\ &= \frac{1}{12}\,(E(X_j) - E(R_j))^2\,, \end{aligned}$$

where we use the assumption that $cov(X_j, X_i) = 0$, and the fact that $cov(UX_j, X_i) = E(U)cov(X_j, X_i) = 0$ and $cov(UX_j, UX_i) = var(U)E(X_j)E(X_i)$ for $j \neq i$, since we assumed that the variables are independent in the original samples. Recall also that $var(U) = \frac{1}{12}$. The variables of the SMOTE samples are therefore slightly positively correlated, unless the distribution of the variables is symmetric or data are high-dimensional, where $E(X_j) = E(R_j)$.

E SMOTE introduces a correlation between samples

The SMOTE samples are correlated with the samples from the minority class that were used to generate their values (X_j and R_j in Eq. 1), and with the other SMOTE samples that were generated using the same minority class samples.

Let S_j^s and S_j^t, $s \neq t$ be the jth variables of two different SMOTE samples, defined as $S_j^s = X_j^s + U^s(R_j^s - X_j^s)$ and $S_j^t = X_j^t + U^t(R_j^t - X_j^t)$, where U^s

and U^t are independent uniform variables $U(0,1)$ and X_j^s, X_j^t, R_j^s and R_j^t are samples from the minority class, defined as in Eq. 1 (note that R_j^s and R_j^t are randomly chosen among the 5 nearest neighbors of X^s and X^r, respectively).

Assuming that the samples of the minority class are independent between each other and have the same variances for the jth variable ($var(X_j^s) = var(X_j)$ for all the samples s of the minority class), the correlation (ρ) of the jth variable between SMOTE samples is

$$\rho\left(S_j^s, S_j^t\right) = \begin{cases} \left(\frac{1}{4}\left(var(X_j) + var(R_j)\right) + \frac{1}{2}cov(R_j, X_j)\right)/var(S_j) & \text{if } (X_j^s = X_j^t \text{ and } R_j^s = R_j^t) \\ & \text{or } (X_j^s = R_j^t \text{ and } X_j^t = R_j^s) \\ \left(\frac{1}{4}var(X_j) + \frac{1}{2}cov(R_j, X_j)\right)/var(S_j) & \text{if } (X_j^s = X_j^t \text{ and } R_j^s \neq R_j^t) \\ \left(\frac{1}{4}var(R_j) + \frac{1}{2}cov(R_j, X_j)\right)/var(S_j) & \text{if } (X_j^s \neq X_j^t \text{ and } R_j^s = R_j^t) \\ 0 & \text{otherwise;} \end{cases}$$

where the $var(S_j)$ is the variance of the SMOTE samples, given in Equations 3, 4 and 6.

Note that the first condition requires that the SMOTE samples were generated using the same two original samples, while the other positive correlations are obtained if the SMOTE samples were generated using exactly one common sample. SMOTE samples are not correlated if they were generated using different original samples.

In the high-dimensional case the correlation simplifies to

$$\rho\left(S_j^s, S_j^t\right) = \begin{cases} 3/4 & \text{if } (X_j^s = X_j^t \text{ and } R_j^s = R_j^t) \text{ or } (X_j^s = R_j^t \text{ and } X_j^t = R_j^s) \\ 3/8 & \text{if } (X_j^s = X_j^t \text{ and } R_j^s \neq R_j^t) \\ 3/8 & \text{if } (X_j^s \neq X_j^t \text{ and } R_j^s = R_j^t) \\ 0 & \text{otherwise.} \end{cases}$$

Using the same assumptions described above, the correlation between a SMOTE sample and an original sample $\mathbf{X}$ for the jth variable is positive if the original sample was used to generate the SMOTE sample, i.e.,

$$\rho(S_j^s, X_j) = \begin{cases} \frac{\sqrt{3}}{2\sqrt{2}} & \text{if } X_j = X_j^s \text{ or } X_j = R_j^s \\ 0 & \text{otherwise.} \end{cases}$$

Proof. We first derive the covariances for the jth variable between two SMOTE samples. The covariance between S_j^s and S_j^t can be expressed as

$$\begin{aligned} cov(S_j^s, S_j^t) &= cov\left(X_j^s + U^s\left(R_j^s - X_j^s\right), X_j^t + U^t\left(R_j^t - X_j^t\right)\right) \\ &= cov(X_j^s, X_j^t) + E(U)cov(X_j^s, R_j^t) - E(U)cov(X_j^s, X_j^t) + \end{aligned}$$

$$
\begin{aligned}
+ &\quad E(U)cov(R_j^s, X_j^t) + E(U)^2 cov(R_j^s, R_j^t) - E(U)^2 cov(R_j^s, X_j^t) - \\
- &\quad E(U)cov(X_j^s, X_j^t) - E(U)^2 cov(X_j^s, R_j^t) + E(U)^2 cov(X_j^s, X_j^t). \\
= &\quad \frac{1}{4}\Big(cov(X_j^s, X_j^t) + cov(X_j^s, R_j^t) + cov(R_j^s, X_j^t) + cov(R_j^s, R_j^t)\Big)
\end{aligned}
$$

Assuming that the samples in the minority class are independent but can be correlated with their nearest neighbors we obtain

$$
cov(S_j^s, S_j^t) = \begin{cases} \frac{1}{4}\left(var(X_j) + var(R_j)\right) + \frac{1}{2}cov(R_j, X_j) & \text{if } (X_j^s = X_j^t \text{ and } R_j^s = R_j^t) \\ & \text{or } (X_j^s = R_j^t \text{ and } X_j^t = R_j^s) \\ \frac{1}{4}var(X_j) + \frac{1}{2}cov(R_j, X_j) & \text{if } (X_j^s = X_j^t \text{ and } R_j^s \neq R_j^t) \\ \frac{1}{4}var(R_j) + \frac{1}{2}cov(R_j, X_j) & \text{if } (X_j^s \neq X_j^t \text{ and } R_j^s = R_j^t) \\ 0 & \text{otherwise.} \end{cases}
$$

For high-dimensional data, where the nearest neighbor and original samples are not correlated and have the same distribution, the result simplifies to

$$
cov(S_j^s, S_j^t) = \begin{cases} \frac{1}{2}var(X_j) & \text{if } (X_j^s = X_j^t \text{ and } R_j^s = R_j^t) \text{ or } (X_j^s = R_j^t \text{ and } X_j^t = R_j^s) \\ \frac{1}{4}var(X_j) & \text{if } (X_j^s = X_j^t \text{ and } R_j^s \neq R_j^t) \\ \frac{1}{4}var(X_j) & \text{if } (X_j^s \neq X_j^t \text{ and } R_j^s = R_j^t) \\ 0 & \text{otherwise.} \end{cases}
$$

The correlations between two variables are derived with the usual formula

$$
\rho(X, Y) = \frac{cov(X, Y)}{\sqrt{var(X)var(Y)}}.
$$

Note that the variance of the SMOTE samples was derived (Equations 3 to 5).

The covariance between a SMOTE sample and original samples in the high-dimensional setting can be derived using the same procedure described above, and is equal to

$$
cov(S_j^s, X_j) = \begin{cases} 1/2 \cdot var(X_j) & \text{if } (X_j = X_j^s \text{ or } X_j = R_j^s) \\ 0 & \text{otherwise,} \end{cases}
$$

therefore the positive correlation is equal to $\frac{\sqrt{3}}{2\sqrt{2}}$.

In practice the correlations tend to be even higher because each original sample has a slightly positive correlation with its nearest neighbor.

F SMOTE reduces the expected Euclidean distance between test samples and the (SMOTE-augmented) minority class, while it increases its variability if the number of variables is large

Let us denote with $d(\mathbf{a},\mathbf{b}) = \sqrt{\sum_{j=1}^{p}(a_j - b_j)^2}$ the Euclidean distance between samples $\mathbf{a}$ and $\mathbf{b}$, with $E(d)$ the expected value of the Euclidean distance and with $var(d)$ its variance; $d^2(\mathbf{a},\mathbf{b}) = \sum_{j=1}^{p}(a_j - b_j)^2$ denotes the Euclidean squared distance. $\mathbf{X^{test}}$ is a sample from the test set, $\mathbf{X}$ is a sample from the original training set and $\mathbf{S}$ is a SMOTE sample. The expected value of the Euclidean distance and its variance are derived using the *Delta method*; as a first step, we derive the expected values and the variances of the Euclidean squared distance ($E(d^2)$ and $var(d^2)$).

Expected value of Euclidean squared distance Assuming that data are high-dimensional and that the original samples are independent and have the same expected values and variances for all the variables ($E(X) = E(X_j^s)$ and $var(X) = var(X_j^s)$ for all j and s), regardless of their class membership (i.e., there are no differences between the classes and all the variables come from distributions that have the same first two moments),

$$E(d^2(\mathbf{X^{test}},\mathbf{X})) = 2p \cdot var(X) > 2p\frac{5}{6} \cdot var(X) = E\left(d^2\left(\mathbf{X^{test}},\mathbf{S}\right)\right) \quad (9)$$

Proof. The expected value of the Euclidean squared distance between a test sample and an original sample can be expressed as

$$\begin{aligned} E(d^2(\mathbf{X^{test}},\mathbf{X})) &= E\left(\sum_{j=1}^{p}(X_j^{test} - X_j^s)^2\right) \\ &= \sum_{j=1}^{p} E\left((X_j^{test} - X_j^s)^2\right) \\ &= \sum_{j=1}^{p}\left(E\left((X_j^{test})^2\right) - 2E(X_j^{test})E(X_j^s) + E\left((X_j^s)^2\right)\right) \\ &= 2 \cdot p \cdot var(X), \quad (10) \end{aligned}$$

which is obtained with simple algebra and using the assumptions of independence between samples and of equality of expected values and variances for all the variables.

The expected value of the Euclidean squared distance between test samples and SMOTE sample is

$$
\begin{aligned}
E(d^2(\mathbf{X^{test}}, \mathbf{S})) &= E\left(\sum_{j=1}^{p}\left(X_j^{test} - (X_j + U(R_j - X_j))\right)^2\right) \\
&= \sum_{j=1}^{p} E\left((X_j^{test} - (X_j + U(R_j - X_j)))^2\right) \\
&= \sum_{j=1}^{p}\left(\frac{4}{3}E(X_j^2) + \frac{1}{3}E(R_j^2) + \frac{1}{3}E(X_jR_j) - E(X_j)^2 - E(X_j)E(R_j)\right) \\
&= p\cdot(\frac{4}{3}var(X_j) + \frac{1}{3}var(R_j) + \frac{1}{3}cov(X_j, R_j) + \\
&\quad + \frac{1}{3}E(X_j)^2 + \frac{1}{3}E(R_j)^2 - \frac{2}{3}E(X_j)E(R_j^{NN})),
\end{aligned}
\tag{11}
$$

which is derived using the same assumptions listed above and with simple algebra.

For symmetric distributions the expression simplifies to

$$E(d^2(\mathbf{X^{test}}, \mathbf{S})) = p\cdot\left(\frac{4}{3}var(X_j) + \frac{1}{3}var(R_j) + \frac{1}{3}cov(X_j, R_j)\right),$$

and for high-dimensional data to

$$E(d^2(\mathbf{X^{test}}, \mathbf{S})) = 2\cdot p\cdot\frac{5}{6}\cdot var(X).$$

Variance of Euclidean squared distance For high-dimensional data, assuming that the samples from the original training set are independent, and that the first four moments of all the p variables are equal for all the samples, if the number of variables is *large* ($p > p^*$) the variance of the Euclidean distance between test samples and samples from the SMOTE-augmented training set is larger if the distance is calculated from a SMOTE sample rather than from an original sample, i.e.,

$$var\left(d^2(\mathbf{X^{test}}, \mathbf{X})\right) < var\left(d^2(\mathbf{X^{test}}, \mathbf{S})\right), \text{if } p > p^*.$$

The minimum number of variables p^* for which the inequality holds depends on the first four moments of the distribution the variables and can be derived

if the distribution of the variables is specified and its first four moments exist and are finite.

Proof. The variance of the Euclidean squared distance between original and test samples can be expressed as

$$\begin{aligned} var\left(d^2(\mathbf{X^{test}}, \mathbf{X})\right) &= var\left(\sum_{j=1}^{p}\left(X_j^{test} - X_j\right)^2\right) \\ &= \sum_{j=1}^{p} E(X_j^{test} - X_j)^4 - 4\sum_{j=1}^{p} var(X_j)^2 \\ &= 2 \cdot p \cdot A - 4 \cdot p \cdot var(X)^2 \\ &= 2 \cdot p \cdot (A - 2 \cdot var(X)^2), \end{aligned} \tag{12}$$

where $A = E(X^4) - 4E(X^3)E(X) + 3E(X^2)^2$; note that the distance variable is not degenerate (has a positive variance) if $A - 2var(X)^2 > 0$.

The variance of the Euclidean squared distance between new and SMOTE samples can be expressed as

$$var\left(d^2(\mathbf{X^{test}}, \mathbf{S})\right) = var\left(\sum_{j=1}^{p}\left(X_j^{test} - (X_j + U(R_j - X_j))\right)^2\right).$$

Defining $Z_j = \left(X_j^{test} - (X_j + U(R_j - X_j))\right)^2$, the variance can be rewritten as

$$\sum_{j=1}^{p} var(Z_j) + 2\sum_{i<j} cov(Z_i, Z_j) = \sum_{j=1}^{p}(E(Z_j^2) - E(Z_j)^2) + 2\sum_{i<j} cov(Z_i, Z_j) \tag{13}$$

With simple algebra and using the assumptions of independence and equality of the first four moments, it can be shown that

$$\begin{aligned} E(Z_j^2) &= \frac{6}{5}E(X^4) + \frac{1}{5}E\left(R^4\right) \\ &- E\left(R^3\right)E(X) + \frac{1}{5}E\left(R^3X\right) - 2E\left(R\right)E(X^3) \\ &+ \frac{1}{5}E(RX^3) - 3E(X)E(X^3) \\ &+ 2E\left(R^2\right)E(X^2) + \frac{1}{5}E\left(R^2X^2\right) + 2E(X^2)E(X^2) \end{aligned}$$

$$+ \quad 2E(RX)E(X^2) - E(R^2X)E(X) - E(RX^2)E(X) \tag{14}$$

and

$$\begin{aligned} cov(Z_i, Z_j) &= \frac{4}{45}E\left(R^2\right)E\left(R^2\right) - \frac{7}{45}E\left(R^2\right)E\left(X^2\right) + \frac{4}{45}E(X^2)E(X^2) \\ &- \frac{1}{45}E\left(R^2\right)E\left(RX\right) - \frac{4}{12}E\left(R^2\right)E\left(R\right)E\left(X\right) \\ &- \frac{1}{45}E\left(X^2\right)E\left(RX\right) + \frac{4}{12}E\left(X^2\right)E\left(R\right)E\left(X\right) \\ &+ \frac{4}{12}E(R)E(X)^3 - \frac{4}{12}E(X)^4 + \frac{2}{90}E\left(RX\right)^2. \end{aligned} \tag{15}$$

These expressions simplify for high-dimensional data to

$$E(Z_j^2) = \frac{7}{10}(2E(X^4) - 8E(X^3)E(X) + 6E(X^2)^2) = \frac{7}{5}A,$$

$$cov(Z_i, Z_j) = \frac{14}{5}var(X)^2 - \left(2\frac{5}{6}var(X)\right)^2 = \frac{1}{45}var(X)^2;$$

Substituting these quantities in Eq. 13, we obtain the variance of the Euclidean squared distance between new and SMOTE samples for high-dimensional data

$$\frac{7}{5}pA - \frac{25}{9}p\cdot var(X)^2 + p(p-1)\frac{1}{45}var(X)^2 = \frac{7}{5}pA + \frac{p^2}{45}var(X)^2 - p\frac{14}{5}var(X)^2.$$

Expected value of Euclidean distance for high-dimensional data For high-dimensional data, making the same assumptions used to derive the variance of the Euclidean squared distance, and using the Delta method with a second order Taylor series approximation, we can derive the approximate expected value of the Euclidean distance as

$$E(d(a,b)) \approx \sqrt{E(d^2(a,b))} - \frac{1}{8}\cdot\frac{var(d^2(a,b))}{E(d^2(a,b))^{3/2}}.$$

The expected value of the Euclidean distance between a test sample and an original sample is approximately

$$E(d(\mathbf{X}^{\mathbf{test}}, \mathbf{X})) \approx \sqrt{2p \cdot var(X)} \left(1 - \frac{1}{16} \cdot \frac{A - 2var(X)^2}{p \cdot var(X)^2}\right),$$

while the expected value of the Euclidean distance between SMOTE and test samples is approximately

$$E(d(\mathbf{X}^{\mathbf{test}}, \mathbf{S})) \approx \sqrt{2p \cdot \frac{5}{6} var(X)} \left(1 - \frac{9}{1000} \frac{7A + \frac{p}{9} var(X)^2 - 14 \cdot var(X)^2}{p \cdot var(X)^2}\right).$$

Let us define δ_E the difference between the two expected values

$$\begin{aligned} \delta_E &= E\left(d(\mathbf{X}^{\mathbf{test}}, \mathbf{X})\right) - E\left(d(\mathbf{X}^{\mathbf{test}}, \mathbf{S})\right) \\ &= \sqrt{2p \cdot var(X)} \left(\frac{1001}{1000} - \sqrt{\frac{5}{6}} + \frac{A - 2var(X)^2}{2000 \cdot p \cdot var(X)^2}\right); \end{aligned} \tag{16}$$

since we assumed that $A - 2var(X)^2 > 0$ (Eq. 13), δ_E is positive for any $p > 0$; moreover, δ_E is an increasing function in p because its first derivative is positive:

$$\frac{\partial \delta_E}{\partial p} = \frac{\left(A - 2var(X)^2\right)\left(125\sqrt{2} - 42\sqrt{15}\right) + 2p \cdot var(X)^2 \left(1000\sqrt{2} - 333\sqrt{15}\right)}{4000\left(var(X)p\right)^{3/2}};$$

it holds that

$$\frac{\partial \delta_E}{\partial p} > 0.$$

Therefore, if there is no difference between the classes and the between samples similarity is evaluated using the Euclidean distance, the test samples are on average more similar to SMOTE samples than to original samples; the difference between the average values increases with the number of variables (p).

Variance of Euclidean distance for high-dimensional data Using the Delta method with a first order Taylor series approximation and the same assumptions used for deriving the expected values, the approximate variance of the Euclidean distance for high-dimensional data is

$$var\left(d(a, b)\right) \approx \frac{var\left(d^2(a, b)\right)}{4 \cdot E\left(d^2(a, b)\right)}.$$

The variance of the Euclidean distance between a test sample and an original sample is approximately

$$var\left(d(\mathbf{X}^{\mathbf{test}},\mathbf{X})\right) \approx \frac{A - 2var(X)^2}{4var(X)}$$

while the variance of the Euclidean distance between SMOTE and test samples is approximately

$$var(d(\mathbf{X}^{\mathbf{test}},\mathbf{S})) \approx \frac{3\cdot\left(7A + \frac{p}{9}var(X)^2 - 14var(X)^2\right)}{100\cdot var(X)}$$

Let us define δ_V the difference between the two variances (SMOTE - original),

$$\begin{aligned}\delta_V &= var\left(d(\mathbf{X}^{\mathbf{test}},\mathbf{S})\right) - var\left(d(\mathbf{X}^{\mathbf{test}},\mathbf{X})\right)\\ &= p\cdot\frac{var(X)}{300} - \frac{A - 2var(X)^2}{25\cdot var(X)},\end{aligned} \tag{17}$$

which is a linear increasing function in p. The function has a root in

$$p^* = \frac{12\,(A - 2var(X)^2)}{var(X)^2}$$

and is positive for any $p > p^*$; p^* is a positive number (because $A - 2var(X)^2 > 0$, see Eq. 12). If more than p^* variables are measured, the variability of the distance is larger when calculated from a SMOTE rather than from an original sample. The value p^* depends on the first four moments of the distribution of the variables, and can be derived if the distribution is known.
For example, if the variables are normally distributed it can be shown that $A = 6var(X)^2$ and $p^* = 48$ (here we used the facts that $E(X^4) = E(X)^4 + 6E(X)^2var(X) + 3var(X)^2$ and $E(X^3) = E(X)^3 + 3E(X)var(X)$ if X is normally distributed). If the variables are uniformly distributed $A = 4.8\cdot var(X)^2$ and $p^* = 33.6$.
