# Supplementary material for: SMOTE for high-dimensional class-imbalanced data
Source: BMC Bioinformatics. 2013 Mar 22;14:106. doi: 10.1186/1471-2105-14-106 (PMC3648438; doi:10.1186/1471-2105-14-106)
Supplement: Additional file 2 — Effect of variable selection on PAM in combination with SMOTE. In the additional file we provide a possible explanation of the effect of variable selection on PAM used with SMOTE. [file 1471-2105-14-106-S2.pdf]

Possible explanation on the effect of variable selection on PAM used with SMOTE

In our simulation studies with high-dimensional class-imbalanced data we observed that under the null case SMOTE had hardly any effect on classification with PAM, when all the $p = 1000$ simulated variables where considered. On the other hand, if only a subset of the variables was used ($G = 40$), SMOTE seemed beneficial in reducing the class-imbalance problem of PAM, decreasing the number of samples classified in the majority class. This behavior can be seen clearly comparing the left and right panels reporting the PAM results in Figure 2.

For example, when the class-imbalance was $k_1^{train} = 0.80$ and variable selection was performed, the PA of the minority class was about 0.25 with SMOTE and about 0.10 if no correction for class-imbalance was used (right panel of Figure 2). On the other hand, SMOTE had no impact on the results if variable selection was not used (PA_1 was about 0.15, regardless of whether SMOTE was used or not (left panel of Figure 2). A similar pattern can be seen also looking at the simulations under the alternative case (Figure 3), where SMOTE changes the classification rule, and improves the predictive accuracy of the minority class, only if the variables are selected.

The classification rule for PAM is based on the distance of the test samples from the shrunken centroids. Values that are more extreme and far from true population values can arise in the minority class; as a consequence in the null case the test samples are on average closer to the shrunken centroid of the majority class; therefore, test samples are more likely to be classified in the majority class. PAM incorporates a class-imbalance correction that has the effect of further increasing the probability of classifying a sample in the majority class. SMOTE does not affect the shrunken centroids (verified by simulation, data not shown), therefore the observed peculiar behavior of SMOTE is only related to its artificial balancing of the training set: the PAM class-imbalance correction is no longer needed when SMOTE is used to obtain a balanced training set, and as a consequence fewer samples are classified in the majority class. We confirmed this finding running a limited set of simulations, where we applied a class-imbalance correction based on the original class-imbalance after using SMOTE, and obtained the same results as with the uncorrected analysis (data not shown).

When variable selection was not performed the average differences of the shrunken centroids were smaller but the distributions of the shrunken centroids overlapped less; for this reason the impact of the class-imbalance correction was small, and SMOTE had a negligible effect when applied on the original data.
