# Supplementary material for: SMOTE for high-dimensional class-imbalanced data
Source: BMC Bioinformatics. 2013 Mar 22;14:106. doi: 10.1186/1471-2105-14-106 (PMC3648438; doi:10.1186/1471-2105-14-106)
Supplement: Additional file 3 — Additional tables for the results obtained on simulated data. In the additional file we report the AUC and G-mean obtained on simulated data. [file 1471-2105-14-106-S3.pdf]

Table 1: **Null hypothesis without variable selection (p=G=1000); AUC .** Area under the ROC curve - AUC (standard deviation) for different levels of class-imbalance (k_1) and classifiers. Results are reported for the original training set (fixed cut-off - NC and adjusted cut-off - CUT-OFF) as well as the SMOTE augmented training set (SMOTE). There is no difference between the classes (null hypothesis) and variable selection is not performed.

k_1		5-NN	DLDA	DQDA	RF	SVM	PAM	PLR-L1	PLR-L2	CART
0.5	NC	0.598	0.606	0.607	0.606	0.603	0.604	0.569	0.607	0.590
		(0.074)	(0.079)	(0.076)	(0.079)	(0.075)	(0.078)	(0.084)	(0.079)	(0.070)
0.4	NC	0.600	0.613	0.608	0.605	0.609	0.612	0.572	0.608	0.587
		(0.074)	(0.082)	(0.081)	(0.079)	(0.078)	(0.080)	(0.086)	(0.081)	(0.069)
	CUT-OFF	0.600	0.613	0.608	0.605	0.609	0.612	0.572	0.608	0.587
		(0.074)	(0.082)	(0.081)	(0.079)	(0.078)	(0.080)	(0.086)	(0.081)	(0.069)
	SMOTE	0.581	0.607	0.605	0.611	0.605	0.607	0.604	0.604	0.588
		(0.065)	(0.081)	(0.076)	(0.079)	(0.080)	(0.079)	(0.082)	(0.079)	(0.069)
0.3	NC	0.600	0.605	0.608	0.607	0.605	0.604	0.563	0.608	0.584
		(0.075)	(0.082)	(0.082)	(0.082)	(0.082)	(0.079)	(0.077)	(0.082)	(0.065)
	CUT-OFF	0.600	0.605	0.608	0.607	0.605	0.604	0.563	0.608	0.584
		(0.075)	(0.082)	(0.082)	(0.082)	(0.082)	(0.079)	(0.077)	(0.082)	(0.065)
	SMOTE	0.557	0.609	0.614	0.608	0.614	0.612	0.608	0.611	0.583
		(0.053)	(0.079)	(0.082)	(0.083)	(0.081)	(0.081)	(0.079)	(0.081)	(0.064)
0.2	NC	0.598	0.604	0.606	0.605	0.602	0.605	0.562	0.605	0.568
		(0.069)	(0.078)	(0.080)	(0.076)	(0.077)	(0.078)	(0.072)	(0.075)	(0.055)
	CUT-OFF	0.598	0.604	0.606	0.605	0.602	0.605	0.562	0.605	0.568
		(0.069)	(0.078)	(0.080)	(0.076)	(0.077)	(0.078)	(0.072)	(0.075)	(0.055)
	SMOTE	0.545	0.609	0.609	0.603	0.610	0.609	0.605	0.611	0.577
		(0.045)	(0.080)	(0.081)	(0.077)	(0.080)	(0.079)	(0.080)	(0.078)	(0.061)
0.1	NC	0.588	0.605	0.605	0.605	0.607	0.606	0.575	0.605	0.554
		(0.069)	(0.079)	(0.077)	(0.080)	(0.079)	(0.077)	(0.084)	(0.077)	(0.047)
	CUT-OFF	0.588	0.605	0.605	0.605	0.607	0.606	0.575	0.605	0.554
		(0.069)	(0.079)	(0.077)	(0.080)	(0.079)	(0.077)	(0.084)	(0.077)	(0.047)
	SMOTE	0.545	0.600	0.603	0.602	0.605	0.601	0.601	0.605	0.555
		(0.043)	(0.076)	(0.078)	(0.075)	(0.080)	(0.078)	(0.079)	(0.079)	(0.048)

Note: The classification rules of the classifiers that appeared in the lower right triangle in the ROC space (that performed worse than random) were negated; the consequence of this was that the AUC for any classifier could not be smaller than 0.5.

Table 2: **Null hypothesis without variable selection (p=G=1000); G-mean.** G-mean (standard deviation) for different levels of class-imbalance (k_1) and classifiers. Results are reported for the original training set (fixed cut-off - NC and adjusted cut-off - CUT-OFF) as well as the SMOTE augmented training set (SMOTE). There is no difference between the classes (null hypothesis) and variable selection is not performed.

k_1		5-NN	DLDA	DQDA	RF	SVM	PAM	PLR-L1	PLR-L2	CART
0.5	NC	0.465	0.490	0.483	0.484	0.461	0.484	0.319	0.485	0.480
		(0.121)	(0.116)	(0.118)	(0.117)	(0.141)	(0.117)	(0.251)	(0.117)	(0.117)
0.4	NC	0.423	0.460	0.435	0.156	0.193	0.451	0.125	0.449	0.470
		(0.150)	(0.132)	(0.138)	(0.181)	(0.218)	(0.130)	(0.200)	(0.137)	(0.135)
	CUT-OFF	0.464	0.460	0.435	0.477	0.485	0.463	0.482	0.465	0.470
		(0.131)	(0.132)	(0.138)	(0.118)	(0.115)	(0.126)	(0.119)	(0.128)	(0.135)
	SMOTE	0.020	0.462	0.340	0.364	0.258	0.462	0.446	0.468	0.480
		(0.078)	(0.128)	(0.174)	(0.170)	(0.189)	(0.123)	(0.160)	(0.122)	(0.127)
0.3	NC	0.279	0.416	0.306	0.004	0.065	0.383	0.042	0.353	0.438
		(0.193)	(0.146)	(0.184)	(0.037)	(0.142)	(0.163)	(0.119)	(0.176)	(0.144)
	CUT-OFF	0.461	0.416	0.306	0.473	0.487	0.429	0.292	0.445	0.438
		(0.125)	(0.146)	(0.184)	(0.131)	(0.115)	(0.147)	(0.255)	(0.142)	(0.144)
	SMOTE	0.003	0.396	0.093	0.157	0.074	0.403	0.425	0.412	0.447
		(0.028)	(0.156)	(0.149)	(0.179)	(0.137)	(0.153)	(0.145)	(0.156)	(0.131)
0.2	NC	0.133	0.324	0.101	0.000	0.016	0.263	0.027	0.195	0.377
		(0.177)	(0.172)	(0.155)	(0.000)	(0.072)	(0.185)	(0.095)	(0.182)	(0.164)
	CUT-OFF	0.472	0.324	0.101	0.471	0.472	0.345	0.307	0.394	0.377
		(0.134)	(0.172)	(0.155)	(0.127)	(0.125)	(0.178)	(0.239)	(0.151)	(0.164)
	SMOTE	0.002	0.284	0.003	0.023	0.007	0.284	0.347	0.286	0.395
		(0.028)	(0.183)	(0.028)	(0.084)	(0.046)	(0.184)	(0.169)	(0.180)	(0.163)
0.1	NC	0.016	0.100	0.000	0.000	0.002	0.052	0.002	0.291	0.244
		(0.070)	(0.157)	(0.000)	(0.000)	(0.024)	(0.122)	(0.025)	(0.209)	(0.191)
	CUT-OFF	0.446	0.100	0.000	0.461	0.460	0.120	0.468	0.405	0.244
		(0.145)	(0.157)	(0.000)	(0.122)	(0.140)	(0.177)	(0.132)	(0.169)	(0.191)
	SMOTE	0.007	0.097	0.000	0.001	0.000	0.101	0.158	0.080	0.290
		(0.046)	(0.153)	(0.000)	(0.014)	(0.000)	(0.155)	(0.179)	(0.147)	(0.182)

Table 3: **Null hypothesis with variable selection (p=1000, G=40); AUC.** Area under the ROC curve - AUC (standard deviation) for different levels of class-imbalance (k_1) and classifiers. Results are reported for the original training set (fixed cut-off - NC and adjusted cut-off - CUT-OFF) as well as the SMOTE augmented training set (SMOTE). There is no difference between the classes (null hypothesis) and variable selection was performed after using SMOTE.

k_1		5-NN	DLDA	DQDA	RF	SVM	PAM	PLR-L1	PLR-L2	CART
0.5	NC	0.602	0.608	0.609	0.607	0.608	0.607	0.605	0.609	0.597
		(0.074)	(0.079)	(0.079)	(0.077)	(0.076)	(0.080)	(0.078)	(0.077)	(0.072)
0.4	NC	0.599	0.603	0.603	0.606	0.603	0.603	0.598	0.601	0.588
		(0.074)	(0.077)	(0.076)	(0.076)	(0.077)	(0.077)	(0.077)	(0.080)	(0.068)
	CUT-OFF	0.599	0.603	0.603	0.606	0.603	0.603	0.598	0.601	0.588
		(0.074)	(0.077)	(0.076)	(0.076)	(0.077)	(0.077)	(0.077)	(0.080)	(0.068)
	SMOTE	0.599	0.602	0.601	0.605	0.600	0.602	0.600	0.598	0.590
		(0.074)	(0.073)	(0.073)	(0.075)	(0.075)	(0.072)	(0.072)	(0.075)	(0.068)
0.3	NC	0.605	0.609	0.609	0.610	0.608	0.611	0.604	0.610	0.583
		(0.074)	(0.081)	(0.079)	(0.079)	(0.078)	(0.081)	(0.079)	(0.082)	(0.070)
	CUT-OFF	0.605	0.609	0.609	0.610	0.608	0.611	0.604	0.610	0.583
		(0.074)	(0.081)	(0.079)	(0.079)	(0.078)	(0.081)	(0.079)	(0.082)	(0.070)
	SMOTE	0.600	0.602	0.602	0.602	0.600	0.604	0.600	0.601	0.593
		(0.075)	(0.079)	(0.076)	(0.076)	(0.078)	(0.076)	(0.074)	(0.076)	(0.070)
0.2	NC	0.597	0.605	0.604	0.612	0.608	0.605	0.603	0.607	0.572
		(0.072)	(0.080)	(0.078)	(0.080)	(0.080)	(0.079)	(0.080)	(0.079)	(0.056)
	CUT-OFF	0.597	0.605	0.604	0.612	0.608	0.605	0.603	0.607	0.572
		(0.072)	(0.080)	(0.078)	(0.080)	(0.080)	(0.079)	(0.080)	(0.079)	(0.056)
	SMOTE	0.606	0.610	0.609	0.607	0.607	0.610	0.609	0.610	0.574
		(0.080)	(0.078)	(0.079)	(0.081)	(0.080)	(0.080)	(0.079)	(0.079)	(0.056)
0.1	NC	0.565	0.601	0.605	0.603	0.600	0.607	0.597	0.601	0.550
		(0.055)	(0.081)	(0.078)	(0.080)	(0.078)	(0.080)	(0.078)	(0.076)	(0.045)
	CUT-OFF	0.565	0.601	0.605	0.603	0.600	0.607	0.597	0.601	0.550
		(0.055)	(0.081)	(0.078)	(0.080)	(0.078)	(0.080)	(0.078)	(0.076)	(0.045)
	SMOTE	0.591	0.604	0.605	0.602	0.605	0.606	0.603	0.606	0.551
		(0.065)	(0.074)	(0.074)	(0.074)	(0.077)	(0.075)	(0.076)	(0.076)	(0.047)

Table 4: **Null hypothesis with variable selection (p=1000, G=40); G-mean.** G-mean (standard deviation) for different levels of class-imbalance (k_1) and classifiers. Results are reported for the original training set (fixed cut-off - NC and adjusted cut-off - CUT-OFF) as well as the SMOTE augmented training set (SMOTE). There is no difference between the classes (null hypothesis) and variable selection was performed after using SMOTE.

k_1		5-NN	DLDA	DQDA	RF	SVM	PAM	PLR-L1	PLR-L2	CART
0.5	NC	0.486	0.488	0.492	0.487	0.482	0.490	0.467	0.489	0.484
		(0.113)	(0.117)	(0.114)	(0.115)	(0.121)	(0.116)	(0.151)	(0.119)	(0.125)
0.4	NC	0.458	0.478	0.473	0.420	0.458	0.442	0.441	0.458	0.471
		(0.134)	(0.122)	(0.121)	(0.152)	(0.136)	(0.138)	(0.159)	(0.133)	(0.129)
	CUT-OFF	0.484	0.478	0.473	0.484	0.479	0.481	0.484	0.483	0.471
		(0.123)	(0.122)	(0.121)	(0.115)	(0.126)	(0.119)	(0.123)	(0.125)	(0.129)
	SMOTE	0.480	0.475	0.468	0.447	0.449	0.476	0.475	0.475	0.475
		(0.121)	(0.116)	(0.115)	(0.135)	(0.129)	(0.116)	(0.118)	(0.121)	(0.125)
0.3	NC	0.323	0.457	0.451	0.252	0.359	0.347	0.367	0.363	0.436
		(0.181)	(0.130)	(0.131)	(0.189)	(0.172)	(0.178)	(0.176)	(0.168)	(0.145)
	CUT-OFF	0.469	0.457	0.451	0.486	0.462	0.463	0.451	0.468	0.436
		(0.127)	(0.130)	(0.131)	(0.119)	(0.124)	(0.123)	(0.145)	(0.126)	(0.145)
	SMOTE	0.480	0.448	0.401	0.365	0.357	0.444	0.435	0.429	0.439
		(0.118)	(0.127)	(0.150)	(0.166)	(0.172)	(0.132)	(0.137)	(0.144)	(0.136)
0.2	NC	0.146	0.410	0.380	0.091	0.228	0.219	0.228	0.207	0.345
		(0.175)	(0.149)	(0.163)	(0.151)	(0.189)	(0.191)	(0.194)	(0.189)	(0.169)
	CUT-OFF	0.444	0.410	0.380	0.477	0.435	0.431	0.397	0.408	0.346
		(0.140)	(0.149)	(0.163)	(0.119)	(0.149)	(0.144)	(0.173)	(0.160)	(0.169)
	SMOTE	0.467	0.394	0.283	0.226	0.187	0.396	0.354	0.342	0.379
		(0.131)	(0.158)	(0.187)	(0.189)	(0.185)	(0.154)	(0.171)	(0.176)	(0.164)
0.1	NC	0.025	0.273	0.204	0.014	0.094	0.078	0.070	0.066	0.226
		(0.086)	(0.177)	(0.183)	(0.065)	(0.151)	(0.142)	(0.138)	(0.132)	(0.189)
	CUT-OFF	0.323	0.273	0.204	0.464	0.364	0.312	0.327	0.288	0.226
		(0.182)	(0.177)	(0.183)	(0.117)	(0.169)	(0.190)	(0.181)	(0.178)	(0.189)
	SMOTE	0.388	0.275	0.068	0.061	0.043	0.268	0.186	0.177	0.223
		(0.162)	(0.189)	(0.135)	(0.128)	(0.110)	(0.191)	(0.189)	(0.187)	(0.185)

Table 5: **Alternative hypothesis with variable selection (p=1000, G=40); AUC.** Area under the ROC curve - AUC (standard deviation) for different levels of class-imbalance (k_1) and classifiers. Results are reported for the original training set (fixed cut-off - NC and adjusted cut-off - CUT-OFF) as well as the SMOTE augmented training set (SMOTE). There is a moderate difference between the classes (alternative hypothesis, $p_{DE} = 20$) and variable selection was performed after using SMOTE.

k_1		5-NN	DLDA	DQDA	RF	SVM	PAM	PLR-L1	PLR-L2	CART
0.5	NC	0.776	0.829	0.824	0.826	0.795	0.844	0.775	0.788	0.675
		(0.107)	(0.100)	(0.101)	(0.102)	(0.108)	(0.095)	(0.113)	(0.110)	(0.101)
0.4	NC	0.759	0.818	0.813	0.811	0.778	0.835	0.765	0.776	0.657
		(0.109)	(0.100)	(0.102)	(0.100)	(0.106)	(0.093)	(0.107)	(0.103)	(0.097)
	CUT-OFF	0.759	0.818	0.813	0.811	0.778	0.835	0.765	0.776	0.657
		(0.109)	(0.100)	(0.102)	(0.100)	(0.106)	(0.093)	(0.107)	(0.103)	(0.097)
	SMOTE	0.767	0.819	0.811	0.810	0.782	0.836	0.771	0.781	0.667
		(0.109)	(0.102)	(0.104)	(0.104)	(0.107)	(0.095)	(0.111)	(0.108)	(0.098)
0.3	NC	0.745	0.814	0.808	0.802	0.773	0.832	0.761	0.773	0.640
		(0.109)	(0.101)	(0.103)	(0.102)	(0.106)	(0.096)	(0.108)	(0.109)	(0.090)
	CUT-OFF	0.745	0.814	0.808	0.802	0.773	0.832	0.761	0.773	0.640
		(0.109)	(0.101)	(0.103)	(0.102)	(0.106)	(0.096)	(0.108)	(0.109)	(0.090)
	SMOTE	0.758	0.816	0.807	0.804	0.772	0.831	0.753	0.763	0.646
		(0.106)	(0.097)	(0.100)	(0.101)	(0.102)	(0.091)	(0.106)	(0.102)	(0.097)
0.2	NC	0.719	0.801	0.795	0.784	0.755	0.819	0.742	0.760	0.626
		(0.106)	(0.108)	(0.110)	(0.112)	(0.112)	(0.104)	(0.117)	(0.113)	(0.088)
	CUT-OFF	0.719	0.801	0.795	0.784	0.755	0.819	0.742	0.760	0.626
		(0.106)	(0.108)	(0.110)	(0.112)	(0.112)	(0.104)	(0.117)	(0.113)	(0.088)
	SMOTE	0.726	0.787	0.773	0.771	0.735	0.799	0.725	0.739	0.617
		(0.111)	(0.105)	(0.111)	(0.106)	(0.110)	(0.109)	(0.112)	(0.109)	(0.081)
0.1	NC	0.631	0.754	0.740	0.725	0.702	0.767	0.708	0.726	0.583
		(0.091)	(0.122)	(0.122)	(0.118)	(0.115)	(0.123)	(0.115)	(0.119)	(0.072)
	CUT-OFF	0.631	0.754	0.740	0.725	0.702	0.767	0.708	0.726	0.583
		(0.091)	(0.122)	(0.122)	(0.118)	(0.115)	(0.123)	(0.115)	(0.119)	(0.072)
	SMOTE	0.682	0.745	0.731	0.731	0.703	0.752	0.695	0.711	0.576
		(0.104)	(0.120)	(0.122)	(0.119)	(0.112)	(0.117)	(0.113)	(0.115)	(0.064)

Table 6: **Alternative hypothesis with variable selection (p=1000, G=40); G-mean.** G-mean (standard deviation) for different levels of class-imbalance (k_1) and classifiers. Results are reported for the original training set (fixed cut-off - NC and adjusted cut-off - CUT-OFF) as well as the SMOTE augmented training set (SMOTE). There is a moderate difference between the classes (alternative hypothesis, $p_{DE} = 20$) and variable selection was performed after using SMOTE.

k_1		5-NN	DLDA	DQDA	RF	SVM	PAM	PLR-L1	PLR-L2	CART
0.5	NC	0.707	0.756	0.753	0.745	0.723	0.757	0.693	0.704	0.657
		(0.110)	(0.102)	(0.104)	(0.104)	(0.113)	(0.102)	(0.113)	(0.109)	(0.119)
0.4	NC	0.674	0.740	0.739	0.712	0.691	0.741	0.667	0.676	0.631
		(0.122)	(0.101)	(0.104)	(0.112)	(0.112)	(0.102)	(0.112)	(0.112)	(0.123)
	CUT-OFF	0.688	0.740	0.739	0.728	0.706	0.744	0.684	0.689	0.631
		(0.113)	(0.101)	(0.104)	(0.107)	(0.110)	(0.101)	(0.109)	(0.110)	(0.123)
	SMOTE	0.708	0.746	0.736	0.722	0.693	0.747	0.682	0.692	0.647
		(0.112)	(0.106)	(0.107)	(0.113)	(0.115)	(0.106)	(0.117)	(0.111)	(0.117)
0.3	NC	0.598	0.737	0.727	0.631	0.626	0.708	0.613	0.616	0.599
		(0.146)	(0.103)	(0.107)	(0.134)	(0.135)	(0.117)	(0.134)	(0.140)	(0.124)
	CUT-OFF	0.684	0.737	0.727	0.723	0.700	0.741	0.672	0.685	0.600
		(0.113)	(0.103)	(0.107)	(0.103)	(0.114)	(0.105)	(0.116)	(0.117)	(0.124)
	SMOTE	0.690	0.734	0.703	0.678	0.624	0.735	0.642	0.647	0.609
		(0.106)	(0.104)	(0.114)	(0.124)	(0.128)	(0.102)	(0.121)	(0.121)	(0.132)
0.2	NC	0.460	0.712	0.696	0.493	0.520	0.648	0.517	0.513	0.545
		(0.172)	(0.120)	(0.127)	(0.178)	(0.173)	(0.141)	(0.159)	(0.162)	(0.154)
	CUT-OFF	0.644	0.712	0.696	0.702	0.669	0.720	0.637	0.651	0.545
		(0.123)	(0.120)	(0.127)	(0.118)	(0.118)	(0.116)	(0.126)	(0.127)	(0.154)
	SMOTE	0.658	0.684	0.593	0.556	0.438	0.687	0.558	0.567	0.531
		(0.113)	(0.128)	(0.149)	(0.160)	(0.185)	(0.126)	(0.154)	(0.154)	(0.153)
0.1	NC	0.126	0.565	0.479	0.153	0.291	0.403	0.223	0.231	0.389
		(0.175)	(0.181)	(0.206)	(0.195)	(0.217)	(0.219)	(0.210)	(0.207)	(0.202)
	CUT-OFF	0.534	0.565	0.479	0.649	0.589	0.598	0.506	0.522	0.389
		(0.171)	(0.181)	(0.206)	(0.120)	(0.156)	(0.177)	(0.190)	(0.181)	(0.202)
	SMOTE	0.569	0.559	0.213	0.206	0.116	0.565	0.354	0.362	0.357
		(0.152)	(0.169)	(0.215)	(0.210)	(0.170)	(0.174)	(0.196)	(0.203)	(0.198)
