# Supplementary figures and images for: SMOTE for high-dimensional class-imbalanced data
Source: BMC Bioinformatics. 2013 Mar 22;14:106. doi: 10.1186/1471-2105-14-106 (PMC3648438; doi:10.1186/1471-2105-14-106)

Analysis without variable selection

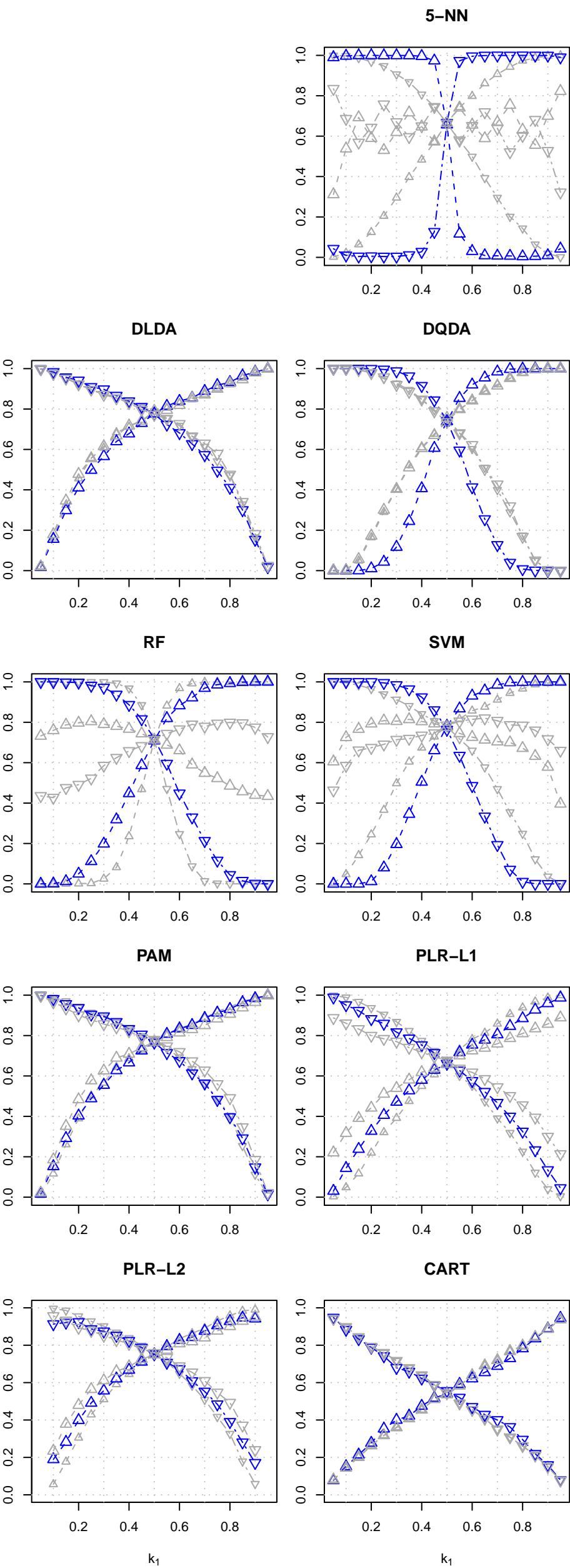

Analysis with variable selection

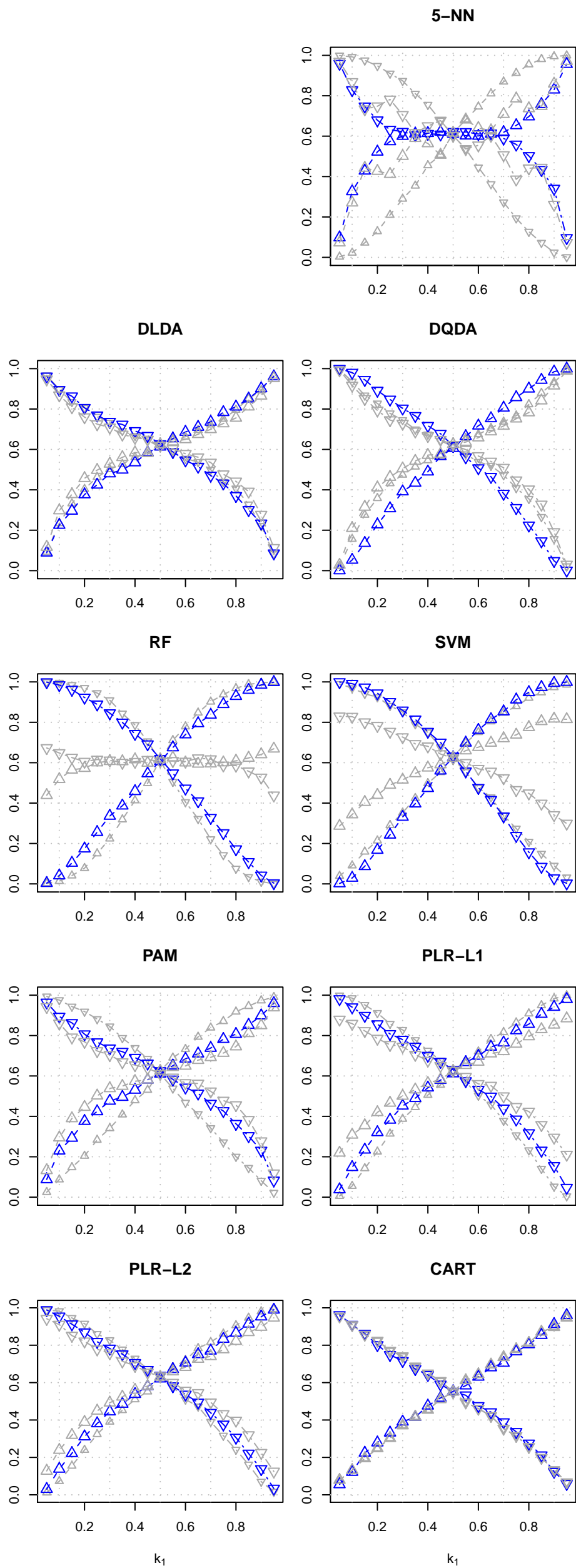

Supplement: Additional file 4 — Results obtained on the data where all variables were differentially expressed. The additional file reports the same information as Figure 3; all variables where differentially expressed (p = pDE = 1, 000). [file 1471-2105-14-106-S4.pdf]
