# Supplementary material for: SMOTE for high-dimensional class-imbalanced data
Source: BMC Bioinformatics. 2013 Mar 22;14:106. doi: 10.1186/1471-2105-14-106 (PMC3648438; doi:10.1186/1471-2105-14-106)

Figure 1: Additional simulation results under the null hypothesis for the data arising from the exponential distribution.

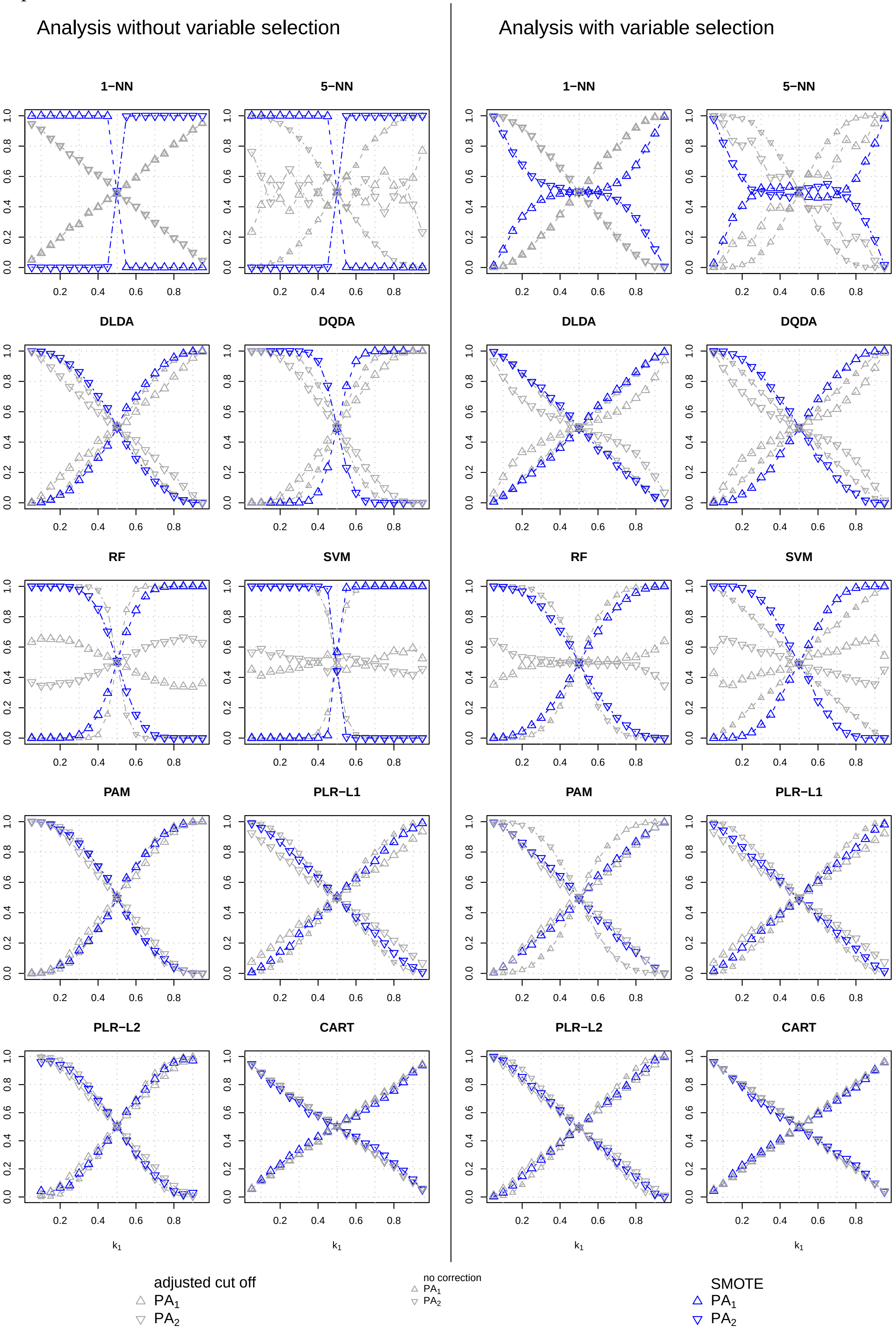

Figure 2: Additional simulation results under the alternative hypothesis for the data arising from the exponential distribution.

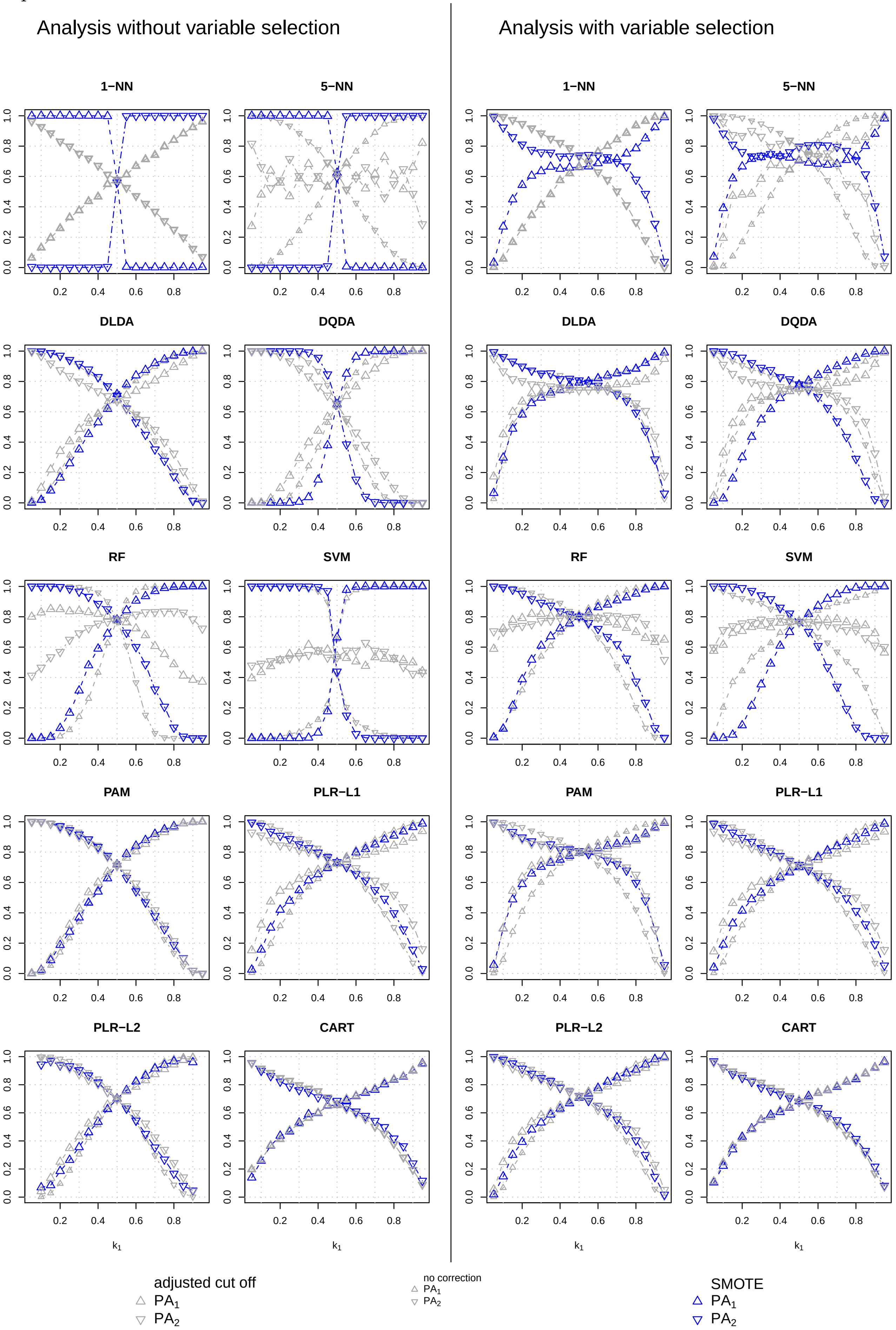

Supplement: Additional file 5 — Results obtained on the data where the variables were simulated from the exponential distribution. The additional file reports the same information as Figure 3 for the setting where variables were simulated from the exponential distribution (page 1 - null case, page 2 - alternative case). [file 1471-2105-14-106-S5.pdf]
