# Supplementary material for: SMOTE for high-dimensional class-imbalanced data
Source: BMC Bioinformatics. 2013 Mar 22;14:106. doi: 10.1186/1471-2105-14-106 (PMC3648438; doi:10.1186/1471-2105-14-106)
Supplement: Additional file 6 — Results obtained on real gene expression data sets. The additional file reports the numerical results obtained by analyzing various gene expression data sets. [file 1471-2105-14-106-S6.pdf]

Table 1: **Performance of the classifiers on the Sotiriou data set with feature selection; prediction of Estrogen receptor status (ER).** Overall predictive accuracy (PA), predictive accuracy for Class 1 (PA_1), predictive accuracy for Class 2 (PA_2), Area under the ROC curve (AUC) and G-mean for 1-NN, 3-NN and 5-NN achieved on the Sotiriou data set for different classifiers and different methods of training set manipulation (no correction - NC, cut-off adjustment (CUT-OFF), SMOTE and undersampling - UNDER). Prediction of Estrogen receptor status (ER). Only 40 variables with the largest absolute value of the t statistic were considered when training the classifiers.

		1-NN	5-NN	DLDA	DQDA	RF	SVM	PAM	PLR-L1	PLR-L2	CART
**NC**	PA	0.879	0.899	0.879	0.889	0.879	0.899	0.919	0.889	0.869	0.838
	PA_1	0.908	0.923	0.892	0.908	0.908	0.923	0.954	0.923	0.908	0.892
	PA_2	0.824	0.853	0.853	0.853	0.824	0.853	0.853	0.824	0.794	0.735
	AUC	0.866	0.894	0.919	0.922	0.917	0.877	0.923	0.901	0.897	0.814
	G-mean	0.865	0.887	0.872	0.880	0.865	0.887	0.902	0.872	0.849	0.810
**CUT-OFF**	PA	0.879	0.889	0.879	0.889	0.879	0.889	0.848	0.828	0.879	0.838
	PA_1	0.908	0.908	0.892	0.908	0.877	0.908	0.846	0.815	0.892	0.892
	PA_2	0.824	0.853	0.853	0.853	0.882	0.853	0.853	0.853	0.853	0.735
	AUC	0.866	0.894	0.905	0.909	0.911	0.878	0.923	0.901	0.918	0.814
	G-mean	0.865	0.880	0.872	0.880	0.880	0.880	0.850	0.834	0.872	0.810
**SMOTE**	PA	0.838	0.877	0.863	0.876	0.880	0.889	0.871	0.838	0.845	0.777
		(0.016)	(0.011)	(0.007)	(0.007)	(0.006)	(0.006)	(0.009)	(0.014)	(0.011)	(0.028)
	PA_1	0.837	0.889	0.868	0.891	0.894	0.908	0.880	0.851	0.869	0.824
		(0.023)	(0.016)	(0.011)	(0.008)	(0.009)	(0.010)	(0.014)	(0.020)	(0.014)	(0.032)
	PA_2	0.839	0.853	0.853	0.846	0.853	0.853	0.853	0.812	0.798	0.686
		(0.016)	(0.000)	(0.000)	(0.013)	(0.000)	(0.000)	(0.000)	(0.021)	(0.022)	(0.049)
	AUC	0.838	0.875	0.914	0.907	0.893	0.867	0.918	0.874	0.871	0.715
		(0.013)	(0.010)	(0.003)	(0.004)	(0.008)	(0.009)	(0.014)	(0.010)	(0.007)	(0.042)
	G-mean	0.838	0.871	0.861	0.868	0.873	0.880	0.866	0.831	0.833	0.751
		(0.013)	(0.008)	(0.005)	(0.008)	(0.005)	(0.005)	(0.007)	(0.013)	(0.012)	(0.032)
**UNDER**	PA	0.846	0.888	0.868	0.874	0.871	0.887	0.862	0.842	0.845	0.783
		(0.016)	(0.007)	(0.009)	(0.011)	(0.009)	(0.006)	(0.011)	(0.022)	(0.017)	(0.033)
	PA_1	0.849	0.907	0.875	0.884	0.880	0.905	0.867	0.841	0.848	0.778
		(0.023)	(0.009)	(0.014)	(0.015)	(0.015)	(0.010)	(0.016)	(0.028)	(0.022)	(0.040)
	PA_2	0.841	0.851	0.855	0.854	0.854	0.852	0.853	0.845	0.839	0.791
		(0.016)	(0.007)	(0.007)	(0.011)	(0.006)	(0.004)	(0.000)	(0.023)	(0.020)	(0.049)
	AUC	0.845	0.880	0.917	0.913	0.910	0.901	0.903	0.895	0.897	0.784
		(0.013)	(0.012)	(0.006)	(0.006)	(0.011)	(0.012)	(0.015)	(0.018)	(0.012)	(0.033)
	G-mean	0.845	0.879	0.865	0.869	0.867	0.878	0.860	0.843	0.844	0.784
		(0.013)	(0.006)	(0.008)	(0.010)	(0.007)	(0.005)	(0.008)	(0.020)	(0.016)	(0.033)

Table 2: **Performance of the classifiers on the Sotiriou data set with feature selection; prediction of histologic grade of the tumor (Grade).** Overall predictive accuracy (PA), predictive accuracy for Class 1 (PA_1), predictive accuracy for Class 2 (PA_2), Area under the ROC curve (AUC) and G-mean for 1-NN, 3-NN and 5-NN achieved on the Sotiriou data set for different classifiers and different methods of training set manipulation (no correction - NC, cut-off adjustment (CUT-OFF), SMOTE and undersampling - UNDER). Prediction of Grade of the tumor (Grade). Only 40 variables with the largest absolute value of the t statistic were considered when training the classifiers.

		1-NN	5-NN	DLDA	DQDA	RF	SVM	PAM	PLR-L1	PLR-L2	CART
**NC**	PA	0.616	0.616	0.677	0.667	0.727	0.636	0.626	0.707	0.677	0.657
	PA_1	0.722	0.722	0.648	0.648	0.759	0.667	0.667	0.741	0.704	0.704
	PA_2	0.489	0.489	0.711	0.689	0.689	0.611	0.578	0.667	0.644	0.600
	AUC	0.606	0.640	0.742	0.730	0.787	0.740	0.649	0.739	0.767	0.619
	G-mean	0.594	0.594	0.679	0.668	0.723	0.638	0.621	0.703	0.673	0.650
**CUT-OFF**	PA	0.616	0.616	0.677	0.667	0.707	0.646	0.626	0.697	0.646	0.657
	PA_1	0.722	0.722	0.648	0.648	0.685	0.648	0.611	0.722	0.648	0.704
	PA_2	0.489	0.489	0.711	0.689	0.733	0.644	0.644	0.667	0.644	0.600
	AUC	0.606	0.640	0.723	0.715	0.778	0.734	0.649	0.744	0.742	0.619
	G-mean	0.594	0.594	0.679	0.668	0.709	0.646	0.628	0.694	0.646	0.650
**SMOTE**	PA	0.628	0.620	0.677	0.660	0.692	0.647	0.641	0.688	0.658	0.641
		(0.035)	(0.025)	(0.015)	(0.017)	(0.017)	(0.017)	(0.018)	(0.013)	(0.015)	(0.034)
	PA_1	0.687	0.667	0.688	0.675	0.711	0.660	0.656	0.713	0.677	0.694
		(0.037)	(0.033)	(0.018)	(0.017)	(0.019)	(0.023)	(0.028)	(0.016)	(0.020)	(0.039)
	PA_2	0.558	0.563	0.663	0.642	0.669	0.637	0.624	0.658	0.635	0.578
		(0.054)	(0.039)	(0.028)	(0.032)	(0.026)	(0.025)	(0.027)	(0.016)	(0.024)	(0.053)
	AUC	0.622	0.689	0.734	0.727	0.760	0.746	0.679	0.752	0.750	0.632
		(0.036)	(0.025)	(0.008)	(0.009)	(0.009)	(0.010)	(0.025)	(0.006)	(0.012)	(0.043)
	G-mean	0.618	0.613	0.675	0.658	0.689	0.648	0.639	0.685	0.656	0.632
		(0.038)	(0.026)	(0.016)	(0.018)	(0.017)	(0.017)	(0.018)	(0.013)	(0.015)	(0.036)
**UNDER**	PA	0.619	0.638	0.676	0.669	0.698	0.661	0.650	0.675	0.680	0.643
		(0.035)	(0.031)	(0.022)	(0.019)	(0.024)	(0.022)	(0.024)	(0.027)	(0.023)	(0.037)
	PA_1	0.685	0.708	0.673	0.660	0.694	0.680	0.656	0.688	0.684	0.655
		(0.037)	(0.045)	(0.024)	(0.023)	(0.029)	(0.026)	(0.031)	(0.033)	(0.028)	(0.052)
	PA_2	0.541	0.554	0.680	0.681	0.702	0.645	0.643	0.659	0.674	0.628
		(0.059)	(0.041)	(0.031)	(0.031)	(0.032)	(0.030)	(0.035)	(0.046)	(0.034)	(0.055)
	AUC	0.613	0.709	0.743	0.741	0.768	0.759	0.699	0.760	0.763	0.640
		(0.037)	(0.023)	(0.012)	(0.012)	(0.011)	(0.011)	(0.023)	(0.021)	(0.014)	(0.043)
	G-mean	0.608	0.625	0.676	0.670	0.698	0.662	0.649	0.673	0.679	0.641
		(0.039)	(0.031)	(0.022)	(0.020)	(0.024)	(0.022)	(0.024)	(0.028)	(0.023)	(0.037)

Table 3: **Performance of the classifiers on the Sotiriou data set without feature selection.** Overall predictive accuracy (PA), predictive accuracy for Class 1 (PA_1), predictive accuracy for Class 2 (PA_2), Area under the ROC curve (AUC) and G-mean for 1-NN, 3-NN and 5-NN achieved on the Sotiriou data set with different methods of training set manipulation (no correction - NC (in brackets we report the results obtained by adjusting the threshold for 5-NN), SMOTE and undersampling - UNDER). Prediction of Estrogen receptor status (**ER**) and Grade of the tumor (**Grade**). All variables were considered when training the classifiers.

		**ER**			**Grade**		
		1-NN	3-NN	5-NN	1-NN	3-NN	5-NN
**NC**	PA	0.788	0.808	0.768 (0.798)	0.616	0.646	0.707 (0.707)
	PA_1	0.877	0.923	0.938 (0.862)	0.704	0.722	0.815 (0.815)
	PA_2	0.618	0.588	0.441 (0.676)	0.511	0.556	0.578 (0.578)
	AUC	0.747	0.809	0.818 (0.818)	0.607	0.685	0.668 (0.668)
	G-mean	0.736	0.737	0.643 (0.763)	0.600	0.633	0.686 (0.686)
**SMOTE**	PA	0.729	0.769	0.789	0.602	0.631	0.671
		(0.022)	(0.019)	(0.016)	(0.033)	(0.027)	(0.027)
	PA_1	0.660	0.706	0.740	0.541	0.545	0.603
		(0.027)	(0.026)	(0.024)	(0.042)	(0.040)	(0.039)
	PA_2	0.859	0.888	0.884	0.674	0.735	0.752
		(0.036)	(0.020)	(0.017)	(0.043)	(0.033)	(0.036)
	AUC	0.760	0.834	0.855	0.608	0.704	0.720
		(0.023)	(0.018)	(0.015)	(0.033)	(0.024)	(0.020)
	G-mean	0.753	0.792	0.808	0.604	0.632	0.673
		(0.023)	(0.017)	(0.015)	(0.033)	(0.028)	(0.028)
**UNDER**	PA	0.786	0.809	0.791	0.620	0.655	0.690
		(0.018)	(0.015)	(0.020)	(0.017)	(0.018)	(0.019)
	PA_1	0.829	0.864	0.866	0.675	0.690	0.760
		(0.020)	(0.018)	(0.016)	(0.021)	(0.021)	(0.026)
	PA_2	0.705	0.704	0.648	0.553	0.614	0.605
		(0.039)	(0.027)	(0.043)	(0.027)	(0.031)	(0.030)
	AUC	0.767	0.837	0.860	0.614	0.689	0.670
		(0.021)	(0.017)	(0.015)	(0.017)	(0.017)	(0.016)
	G-mean	0.764	0.780	0.748	0.611	0.651	0.678
		(0.022)	(0.018)	(0.027)	(0.017)	(0.019)	(0.020)

Table 4: **Performance of the classifiers on the Pittman data set with feature selection; prediction of Estrogen receptor status (ER).** Overall predictive accuracy (PA), predictive accuracy for Class 1 (PA_1), predictive accuracy for Class 2 (PA_2), Area under the ROC curve (AUC) and G-mean for 1-NN, 3-NN and 5-NN achieved on the Pittman data set for different classifiers and different methods of training set manipulation (no correction - NC, cut-off adjustment (CUT-OFF), SMOTE and undersampling - UNDER). Prediction of Estrogen receptor status (ER). Only 40 variables with the largest absolute value of the t statistic were considered when training the classifiers.

		1-NN	5-NN	DLDA	DQDA	RF	SVM	PAM	PLR-L1	PLR-L2	CART
**NC**	PA	0.823	0.848	0.861	0.861	0.873	0.861	0.886	0.816	0.791	0.785
	PA_1	0.882	0.873	0.864	0.864	0.909	0.900	0.900	0.873	0.864	0.845
	PA_2	0.688	0.792	0.854	0.854	0.792	0.771	0.854	0.688	0.625	0.646
	AUC	0.785	0.862	0.906	0.902	0.879	0.863	0.885	0.858	0.848	0.601
	G-mean	0.779	0.831	0.859	0.859	0.848	0.833	0.877	0.775	0.735	0.739
**CUT-OFF**	PA	0.823	0.823	0.861	0.861	0.842	0.873	0.861	0.842	0.842	0.785
	PA_1	0.882	0.809	0.864	0.864	0.836	0.891	0.864	0.855	0.855	0.845
	PA_2	0.688	0.854	0.854	0.854	0.854	0.833	0.854	0.812	0.812	0.646
	AUC	0.785	0.862	0.907	0.907	0.879	0.863	0.886	0.862	0.857	0.601
	G-mean	0.779	0.831	0.859	0.859	0.845	0.862	0.859	0.833	0.833	0.739
**SMOTE**	PA	0.798	0.828	0.859	0.867	0.880	0.863	0.870	0.826	0.821	0.818
		(0.017)	(0.014)	(0.005)	(0.007)	(0.006)	(0.009)	(0.008)	(0.009)	(0.012)	(0.019)
	PA_1	0.815	0.813	0.866	0.879	0.902	0.900	0.887	0.860	0.861	0.855
		(0.021)	(0.018)	(0.007)	(0.009)	(0.007)	(0.007)	(0.010)	(0.005)	(0.012)	(0.024)
	PA_2	0.759	0.862	0.845	0.840	0.829	0.777	0.832	0.747	0.728	0.732
		(0.023)	(0.017)	(0.010)	(0.010)	(0.015)	(0.020)	(0.014)	(0.029)	(0.029)	(0.034)
	AUC	0.787	0.881	0.906	0.903	0.896	0.882	0.890	0.847	0.867	0.777
		(0.017)	(0.009)	(0.003)	(0.003)	(0.006)	(0.008)	(0.011)	(0.008)	(0.009)	(0.031)
	G-mean	0.786	0.837	0.855	0.860	0.865	0.836	0.859	0.801	0.791	0.791
		(0.017)	(0.013)	(0.006)	(0.007)	(0.008)	(0.012)	(0.009)	(0.015)	(0.017)	(0.021)
**UNDER**	PA	0.790	0.848	0.851	0.852	0.857	0.849	0.858	0.839	0.816	0.767
		(0.019)	(0.013)	(0.009)	(0.010)	(0.012)	(0.016)	(0.010)	(0.014)	(0.020)	(0.020)
	PA_1	0.792	0.851	0.852	0.854	0.860	0.852	0.866	0.845	0.815	0.772
		(0.027)	(0.017)	(0.012)	(0.012)	(0.015)	(0.020)	(0.014)	(0.018)	(0.024)	(0.025)
	PA_2	0.786	0.843	0.846	0.848	0.851	0.841	0.839	0.826	0.817	0.755
		(0.039)	(0.023)	(0.012)	(0.013)	(0.017)	(0.018)	(0.016)	(0.035)	(0.038)	(0.040)
	AUC	0.789	0.881	0.905	0.904	0.901	0.885	0.889	0.887	0.872	0.753
		(0.020)	(0.012)	(0.006)	(0.005)	(0.008)	(0.010)	(0.010)	(0.013)	(0.016)	(0.026)
	G-mean	0.789	0.846	0.849	0.851	0.855	0.846	0.852	0.835	0.816	0.763
		(0.021)	(0.014)	(0.009)	(0.010)	(0.011)	(0.014)	(0.010)	(0.018)	(0.022)	(0.022)

Table 5: **Performance of the classifiers on the Pittman data set with feature selection; prediction of nuclear Grade (Grade).** Overall predictive accuracy (PA), predictive accuracy for Class 1 (PA_1), predictive accuracy for Class 2 (PA_2), Area under the ROC curve (AUC) and G-mean for 1-NN, 3-NN and 5-NN achieved on the Pittman data set for different classifiers and different methods of training set manipulation (no correction - NC, cut-off adjustment (CUT-OFF), SMOTE and undersampling - UNDER). Prediction of nuclear Grade (Grade). Only 40 variables with the largest absolute value of the t statistic were considered when training the classifiers.

		1-NN	5-NN	DLDA	DQDA	RF	SVM	PAM	PLR-L1	PLR-L2	CART
**NC**	PA	0.709	0.722	0.804	0.804	0.823	0.766	0.778	0.753	0.728	0.696
	PA_1	0.789	0.800	0.811	0.811	0.874	0.789	0.832	0.821	0.789	0.695
	PA_2	0.587	0.603	0.794	0.794	0.746	0.730	0.698	0.651	0.635	0.698
	AUC	0.688	0.771	0.861	0.861	0.849	0.804	0.833	0.786	0.779	0.589
	G-mean	0.681	0.695	0.802	0.802	0.807	0.759	0.762	0.731	0.708	0.697
**CUT-OFF**	PA	0.709	0.766	0.797	0.804	0.778	0.747	0.753	0.734	0.772	0.696
	PA_1	0.789	0.800	0.800	0.811	0.768	0.758	0.737	0.726	0.779	0.695
	PA_2	0.587	0.714	0.794	0.794	0.794	0.730	0.778	0.746	0.762	0.698
	AUC	0.688	0.771	0.852	0.849	0.852	0.807	0.833	0.784	0.816	0.589
	G-mean	0.681	0.756	0.797	0.802	0.781	0.744	0.757	0.736	0.770	0.697
**SMOTE**	PA	0.738	0.765	0.799	0.801	0.816	0.785	0.799	0.761	0.747	0.705
		(0.019)	(0.021)	(0.011)	(0.010)	(0.013)	(0.014)	(0.012)	(0.011)	(0.017)	(0.025)
	PA_1	0.762	0.763	0.800	0.809	0.850	0.813	0.801	0.781	0.783	0.728
		(0.027)	(0.030)	(0.014)	(0.013)	(0.016)	(0.018)	(0.017)	(0.013)	(0.023)	(0.029)
	PA_2	0.701	0.768	0.797	0.788	0.764	0.741	0.797	0.732	0.693	0.670
		(0.031)	(0.028)	(0.019)	(0.020)	(0.020)	(0.021)	(0.018)	(0.016)	(0.027)	(0.038)
	AUC	0.731	0.812	0.858	0.858	0.852	0.818	0.847	0.794	0.779	0.655
		(0.020)	(0.015)	(0.005)	(0.005)	(0.006)	(0.009)	(0.010)	(0.005)	(0.011)	(0.032)
	G-mean	0.730	0.765	0.799	0.798	0.806	0.776	0.799	0.756	0.736	0.698
		(0.020)	(0.020)	(0.012)	(0.011)	(0.013)	(0.014)	(0.012)	(0.011)	(0.018)	(0.026)
**UNDER**	PA	0.733	0.774	0.798	0.800	0.798	0.784	0.797	0.779	0.754	0.713
		(0.024)	(0.019)	(0.013)	(0.014)	(0.016)	(0.014)	(0.016)	(0.019)	(0.019)	(0.030)
	PA_1	0.756	0.792	0.799	0.799	0.800	0.787	0.802	0.784	0.756	0.702
		(0.029)	(0.024)	(0.020)	(0.021)	(0.024)	(0.020)	(0.024)	(0.025)	(0.023)	(0.046)
	PA_2	0.698	0.747	0.797	0.802	0.795	0.778	0.791	0.771	0.751	0.730
		(0.038)	(0.031)	(0.020)	(0.019)	(0.022)	(0.021)	(0.023)	(0.031)	(0.035)	(0.044)
	AUC	0.727	0.816	0.862	0.863	0.857	0.837	0.844	0.834	0.815	0.683
		(0.025)	(0.013)	(0.007)	(0.008)	(0.007)	(0.011)	(0.015)	(0.012)	(0.014)	(0.037)
	G-mean	0.726	0.769	0.798	0.800	0.797	0.783	0.796	0.777	0.754	0.715
		(0.026)	(0.020)	(0.013)	(0.013)	(0.015)	(0.014)	(0.016)	(0.020)	(0.020)	(0.029)

Table 6: **Performance of the classifiers on the Pittman data set without feature selection.** Overall predictive accuracy (PA), predictive accuracy for Class 1 (PA_1), predictive accuracy for Class 2 (PA_2), Area under the ROC curve (AUC) and G-mean for 1-NN, 3-NN and 5-NN achieved on the Pittman data set with different methods of training set manipulation (no correction - NC (in brackets we report the results obtained by adjusting the threshold for 5-NN), SMOTE and undersampling - UNDER). Prediction of Estrogen receptor status (**ER**) and Grade of the tumor (**Grade**). All variables were considered when training the classifiers.

		**ER**			**Grade**		
		1-NN	3-NN	5-NN	1-NN	3-NN	5-NN
**NC**	PA	0.772	0.823	0.848 (0.829)	0.601	0.715	0.728 (0.785)
	PA_1	0.891	0.945	0.936 (0.855)	0.663	0.737	0.747 (0.747)
	PA_2	0.500	0.542	0.646 (0.771)	0.508	0.683	0.698 (0.841)
	AUC	0.695	0.820	0.852 (0.852)	0.586	0.744	0.748 (0.748)
	G-mean	0.667	0.716	0.778 (0.812)	0.580	0.709	0.722 (0.793)
**SMOTE**	PA	0.438	0.397	0.396	0.457	0.474	0.466
		(0.016)	(0.014)	(0.016)	(0.016)	(0.015)	(0.014)
	PA_1	0.241	0.161	0.149	0.166	0.144	0.125
		(0.021)	(0.019)	(0.022)	(0.023)	(0.023)	(0.021)
	PA_2	0.889	0.938	0.962	0.896	0.971	0.979
		(0.020)	(0.016)	(0.016)	(0.021)	(0.015)	(0.014)
	AUC	0.565	0.628	0.688	0.531	0.574	0.619
		(0.014)	(0.016)	(0.018)	(0.015)	(0.015)	(0.016)
	G-mean	0.462	0.389	0.378	0.385	0.373	0.349
		(0.020)	(0.023)	(0.029)	(0.027)	(0.030)	(0.031)
**UNDER**	PA	0.757	0.808	0.821	0.633	0.778	0.792
		(0.020)	(0.021)	(0.021)	(0.038)	(0.024)	(0.028)
	PA_1	0.797	0.838	0.835	0.599	0.685	0.697
		(0.023)	(0.025)	(0.024)	(0.021)	(0.018)	(0.016)
	PA_2	0.665	0.737	0.790	0.577	0.624	0.634
		(0.040)	(0.037)	(0.032)	(0.029)	(0.026)	(0.024)
	AUC	0.731	0.838	0.854	0.605	0.730	0.757
		(0.023)	(0.016)	(0.016)	(0.022)	(0.018)	(0.016)
	G-mean	0.728	0.786	0.812	0.604	0.696	0.708
		(0.024)	(0.023)	(0.022)	(0.022)	(0.018)	(0.016)

Table 7: **Performance of the classifiers on the Miller data set with feature selection; prediction of Estrogen receptor status (ER).** Overall predictive accuracy (PA), predictive accuracy for Class 1 (PA_1), predictive accuracy for Class 2 (PA_2), Area under the ROC curve (AUC) and G-mean for 1-NN, 3-NN and 5-NN achieved on the Miller data set for different classifiers and different methods of training set manipulation (no correction - NC, cut-off adjustment (CUT-OFF), SMOTE and undersampling - UNDER). Prediction of Estrogen receptor status (ER). Only 40 variables with the largest absolute value of the t statistic were considered when training the classifiers.

		1-NN	5-NN	DLDA	DQDA	RF	SVM	PAM	PLR-L1	PLR-L2	CART
**NC**	PA	0.846	0.870	0.907	0.903	0.883	0.891	0.911	0.887	0.846	0.891
	PA_1	0.897	0.920	0.915	0.911	0.939	0.906	0.925	0.441	0.930	0.953
	PA_2	0.529	0.559	0.853	0.853	0.529	0.794	0.824	0.958	0.324	0.500
	AUC	0.713	0.889	0.901	0.901	0.876	0.854	0.906	0.906	0.881	0.565
	G-mean	0.689	0.717	0.884	0.881	0.705	0.848	0.873	0.650	0.548	0.690
**CUT-OFF**	PA	0.846	0.838	0.903	0.899	0.874	0.899	0.838	0.879	0.887	0.887
	PA_1	0.897	0.826	0.911	0.906	0.869	0.906	0.831	0.878	0.883	0.939
	PA_2	0.529	0.912	0.853	0.853	0.912	0.853	0.882	0.882	0.912	0.559
	AUC	0.713	0.889	0.904	0.905	0.882	0.857	0.906	0.903	0.893	0.565
	G-mean	0.689	0.868	0.881	0.879	0.890	0.879	0.856	0.880	0.897	0.724
**SMOTE**	PA	0.831	0.838	0.893	0.893	0.892	0.886	0.887	0.862	0.864	0.846
		(0.010)	(0.010)	(0.002)	(0.003)	(0.008)	(0.006)	(0.004)	(0.007)	(0.008)	(0.011)
	PA_1	0.841	0.830	0.895	0.899	0.915	0.903	0.888	0.886	0.885	0.882
		(0.010)	(0.011)	(0.003)	(0.003)	(0.006)	(0.005)	(0.005)	(0.006)	(0.008)	(0.012)
	PA_2	0.766	0.888	0.882	0.856	0.748	0.779	0.882	0.715	0.734	0.619
		(0.042)	(0.019)	(0.000)	(0.009)	(0.037)	(0.022)	(0.000)	(0.034)	(0.040)	(0.050)
	AUC	0.804	0.876	0.906	0.905	0.873	0.875	0.906	0.824	0.873	0.730
		(0.021)	(0.007)	(0.002)	(0.002)	(0.006)	(0.006)	(0.011)	(0.014)	(0.006)	(0.045)
	G-mean	0.802	0.858	0.889	0.877	0.827	0.839	0.885	0.796	0.805	0.738
		(0.022)	(0.011)	(0.001)	(0.005)	(0.021)	(0.012)	(0.002)	(0.019)	(0.022)	(0.029)
**UNDER**	PA	0.788	0.878	0.879	0.881	0.866	0.882	0.881	0.851	0.840	0.761
		(0.018)	(0.008)	(0.006)	(0.007)	(0.009)	(0.006)	(0.006)	(0.012)	(0.014)	(0.024)
	PA_1	0.784	0.878	0.880	0.881	0.864	0.881	0.881	0.884	0.836	0.761
		(0.019)	(0.009)	(0.007)	(0.008)	(0.010)	(0.006)	(0.007)	(0.024)	(0.016)	(0.024)
	PA_2	0.809	0.877	0.878	0.879	0.880	0.884	0.880	0.846	0.865	0.765
		(0.051)	(0.024)	(0.015)	(0.014)	(0.017)	(0.016)	(0.014)	(0.013)	(0.037)	(0.061)
	AUC	0.797	0.891	0.904	0.905	0.889	0.878	0.903	0.892	0.891	0.761
		(0.028)	(0.011)	(0.007)	(0.005)	(0.011)	(0.011)	(0.012)	(0.015)	(0.013)	(0.035)
	G-mean	0.796	0.877	0.879	0.880	0.872	0.882	0.880	0.864	0.850	0.762
		(0.028)	(0.012)	(0.008)	(0.008)	(0.009)	(0.008)	(0.008)	(0.014)	(0.020)	(0.034)

Table 8: **Performance of the classifiers on the Miller data set with feature selection; prediction of histologic grade of the tumor (Grade).** Overall predictive accuracy (PA), predictive accuracy for Class 1 (PA_1), predictive accuracy for Class 2 (PA_2), Area under the ROC curve (AUC) and G-mean for 1-NN, 3-NN and 5-NN achieved on the Miller data set for different classifiers and different methods of training set manipulation (no correction - NC, cut-off adjustment (CUT-OFF), SMOTE and undersampling - UNDER). Prediction of Grade of the tumor (Grade). Only 40 variables with the largest absolute value of the t statistic were considered when training the classifiers.

		1-NN	5-NN	DLDA	DQDA	RF	SVM	PAM	PLR-L1	PLR-L2	CART
**NC**	PA	0.811	0.831	0.851	0.835	0.855	0.855	0.863	0.851	0.827	0.859
	PA_1	0.877	0.903	0.851	0.831	0.903	0.856	0.877	0.928	0.897	0.918
	PA_2	0.574	0.574	0.852	0.852	0.685	0.852	0.815	0.574	0.574	0.648
	AUC	0.725	0.863	0.888	0.887	0.881	0.863	0.900	0.883	0.875	0.658
	G-mean	0.710	0.720	0.852	0.841	0.786	0.854	0.845	0.730	0.718	0.771
**CUT-OFF**	PA	0.811	0.863	0.847	0.823	0.843	0.863	0.847	0.847	0.847	0.863
	PA_1	0.877	0.882	0.841	0.815	0.826	0.882	0.841	0.841	0.846	0.918
	PA_2	0.574	0.796	0.870	0.852	0.907	0.796	0.870	0.870	0.852	0.667
	AUC	0.725	0.863	0.882	0.883	0.882	0.878	0.900	0.889	0.888	0.658
	G-mean	0.710	0.838	0.856	0.833	0.866	0.838	0.856	0.856	0.849	0.782
**SMOTE**	PA	0.797	0.805	0.826	0.831	0.848	0.836	0.831	0.834	0.823	0.801
		(0.016)	(0.010)	(0.005)	(0.005)	(0.008)	(0.009)	(0.009)	(0.009)	(0.012)	(0.017)
	PA_1	0.804	0.789	0.815	0.823	0.878	0.838	0.821	0.858	0.842	0.837
		(0.015)	(0.013)	(0.005)	(0.005)	(0.006)	(0.011)	(0.009)	(0.008)	(0.013)	(0.018)
	PA_2	0.774	0.861	0.866	0.860	0.739	0.827	0.868	0.745	0.755	0.670
		(0.045)	(0.024)	(0.014)	(0.013)	(0.026)	(0.018)	(0.022)	(0.021)	(0.026)	(0.048)
	AUC	0.789	0.858	0.888	0.885	0.871	0.858	0.877	0.870	0.855	0.738
		(0.024)	(0.009)	(0.001)	(0.001)	(0.004)	(0.005)	(0.011)	(0.005)	(0.007)	(0.039)
	G-mean	0.788	0.824	0.840	0.841	0.805	0.832	0.844	0.800	0.797	0.748
		(0.025)	(0.012)	(0.007)	(0.007)	(0.015)	(0.010)	(0.012)	(0.013)	(0.016)	(0.027)
**UNDER**	PA	0.785	0.823	0.829	0.828	0.831	0.830	0.829	0.820	0.821	0.775
		(0.017)	(0.012)	(0.009)	(0.009)	(0.011)	(0.009)	(0.011)	(0.015)	(0.011)	(0.017)
	PA_1	0.792	0.815	0.814	0.812	0.824	0.818	0.815	0.813	0.815	0.779
		(0.017)	(0.015)	(0.011)	(0.011)	(0.013)	(0.010)	(0.012)	(0.016)	(0.012)	(0.019)
	PA_2	0.760	0.853	0.881	0.889	0.858	0.875	0.881	0.844	0.843	0.762
		(0.046)	(0.031)	(0.019)	(0.019)	(0.021)	(0.022)	(0.022)	(0.035)	(0.030)	(0.045)
	AUC	0.776	0.864	0.887	0.887	0.879	0.877	0.879	0.878	0.870	0.723
		(0.025)	(0.008)	(0.004)	(0.005)	(0.008)	(0.008)	(0.011)	(0.012)	(0.011)	(0.037)
	G-mean	0.775	0.834	0.847	0.849	0.840	0.846	0.847	0.828	0.829	0.770
		(0.025)	(0.016)	(0.010)	(0.010)	(0.013)	(0.012)	(0.013)	(0.019)	(0.016)	(0.024)
